# Occurrence of 97 Pharmaceuticals in Wastewater and Receiving Waters: Analytical Validation and Treatment Influence

**DOI:** 10.3390/jox15030078

**Published:** 2025-05-23

**Authors:** Paula Paíga, Sónia Figueiredo, Manuela Correia, Magda André, Roberto Barbosa, Sandra Jorge, Cristina Delerue-Matos

**Affiliations:** 1 REQUIMTE/LAQV, Instituto Superior de Engenharia do Porto, Instituto Politécnico do Porto, Rua Dr. António Bernardino de Almeida, 431, 4249-015 Porto, Portugal; mmb@isep.ipp.pt (M.C.); cmm@isep.ipp.pt (C.D.-M.); 2 Águas do Centro Litoral, SA, Grupo Águas de Portugal, ETA da Boavista, Avenida Dr. Luís Albuquerque, 3030-410 Coimbra, Portugal; magda.almeida@adp.pt (M.A.); rbarbosa@adp.pt (R.B.); s.jorge@adp.pt (S.J.)

**Keywords:** pharmaceutical contamination, seasonal variability, surface water, micropollutants, wastewater samples

## Abstract

This study analyzed 97 pharmaceuticals in samples of surface water, as well as influent and effluent from various wastewater treatment plants (WWTPs), during winter 2022 and spring 2023. Approximately 40% of the tested compounds were detected, at amounts ranging from below the methods’ detection limits to 5623 ng/L (2-hydroxyibuprofen in surface water) and 12,664 ng/L (caffeine in wastewater). Twelve compounds (acetaminophen, ampicillin, azithromycin, caffeine, fluoxetine, gemfibrozil, 2-hydroxyibuprofen, ibuprofen, ketoprofen, mazindol, naproxen, and salicylic acid) were detected with a 100% frequency in both surface water and wastewater samples. The observed high detection frequency of pharmaceuticals within the nonsteroidal anti-inflammatory drugs/analgesics, antibiotics, and psychiatric drug classes aligns with their high consumption. Caffeine was both the compound with the highest concentration and the most prevalent compound detected. Seasonal differences were observed, with higher concentrations detected during winter. Six of the eleven targeted metabolites and degradation products were detected in at least one sample. Risk quotient assessment revealed potential ecological risks, particularly for atorvastatin, caffeine, carbamazepine, and venlafaxine, exceeding risk thresholds for various trophic levels. The studied WWTPs showed limited removal efficiencies, with some compounds presenting higher concentrations in effluent than in influent, emphasizing the need for enhanced treatment to mitigate micropollutant risks.

## 1. Introduction

The significant advancements in and growing availability of medications for treating various conditions and diseases have been affecting the environment [1]. The environmental impact of pharmaceuticals is not just a local concern, but a global problem affecting ecosystems worldwide [2,3]. The persistence and accumulation of pharmaceutical compounds in aquatic environments pose significant ecological and public health risks [4]. While the benefits of pharmaceuticals in both human and veterinary medicine have been established, these compounds are continuously released into natural water bodies [1]. Wastewater treatment plants (WWTPs) are considered a significant pathway for pharmaceutical discharge, highlighting their limitations in removing such contaminants [1]. Therefore, there is an urgent need for comprehensive monitoring studies to assess not only the presence and levels of pharmaceuticals in the environment but also their detection frequency and the various pathways by which they enter water systems. Such studies are essential for understanding the effects of pharmaceutical pollution and for developing effective strategies to reduce its impacts on ecosystems and human health.

Advancements in analytical techniques, such as ultra-high-performance liquid chromatography coupled with tandem mass spectrometry (UHPLC-MS/MS), have been widely employed to enhance detection at very low concentration levels (ng/L), allowing for more precise identification and quantification of these pollutants, including metabolites and degradation products [5,6,7,8,9,10,11,12,13]. The scientific community has significantly increased the number of studies worldwide on the detection of pharmaceuticals in various environmental matrices, while also developing strategies for their removal. In 2022, Hatami et al. [14] emphasized the growing demand for technical support in handling large datasets. Moreover, Bukar et al. [15] mentioned that the increasing complexity of database information requires advanced data management and visualization tools. Bibliometric analysis has emerged as a crucial method for identifying research trends and has significantly benefited from recent advancements [14,15,16]. Among the various tools available, VOSviewer has emerged as a powerful open-source software for bibliometric mapping [15]. It can efficiently process extensive datasets and extract bibliographic networks with insightful visualizations [15,16]. Compatible with data sources such as WoS, Scopus, WDimensions, PubMed, and RIS format files, VOSviewer enables researchers to analyze the scientific literature, and its software offers an intuitive platform for creating and exploring network-based maps, making it an essential tool for bibliometric research [15].

**Figure 1:**
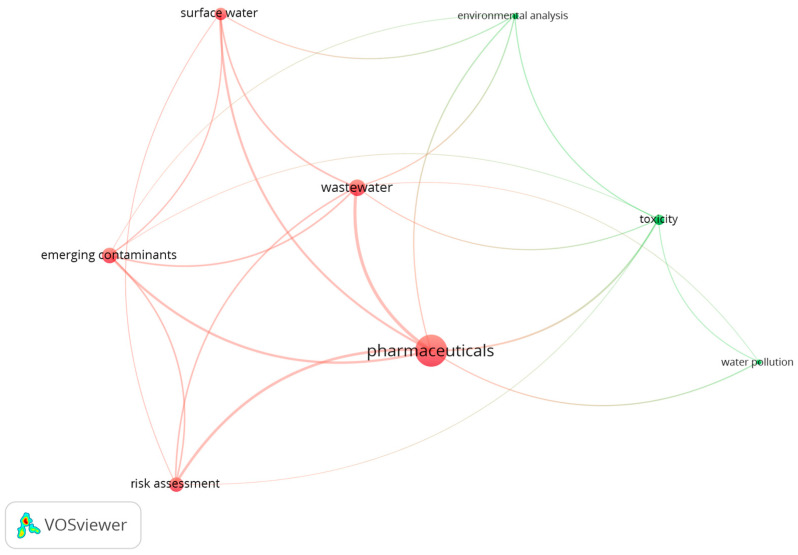
Network diagram generated using the VOSviewer program, based on a search using 8 specific keywords related to pharmaceuticals in the environment.

**Figure 2:**
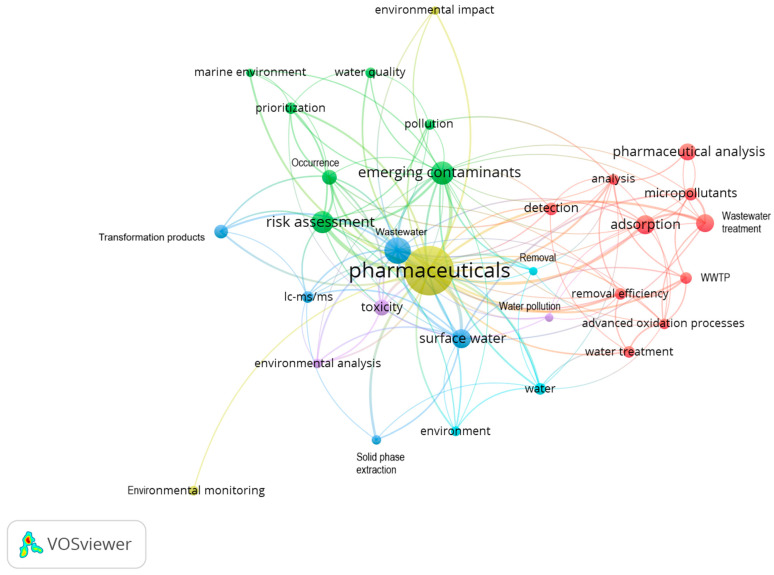
Network diagram generated using the VOSviewer program, based on a search using 32 keywords related to pharmaceuticals in the environment.

To explore the environmental impacts of pharmaceuticals by using the VOSviewer software version 1.6.20, a literature search was conducted with the keywords ‘pharmaceuticals’ and ‘environment’. The resulting database (656 published articles) was analyzed, and network diagrams were generated. The keywords employed to generate the network shown in Figure 1 are ‘emerging contaminants’, ‘environmental analysis’, ‘pharmaceuticals’, ‘risk assessment’, ‘surface water’, ‘toxicity’, ‘wastewater’, and ‘water pollution’. These keywords were selected based on their direct relevance to this research, which investigates the environmental impact of pharmaceuticals within aquatic systems. The network diagram in Figure 2 highlights the 32 most frequent keywords within the published studies. The network diagrams show the co-occurrence relationships between the pharmaceutical-related keywords and their environmental implications. Nodes represent key terms, their size reflects occurrence frequency, and the links indicate their interconnections. Different colors represent distinct research clusters.

Analysis of the network in Figure 1 presents ‘pharmaceuticals’ as the central theme, with strong connections to key areas such as risk assessment, emerging contaminants, and various water environments. The network is organized into two distinct clusters: Cluster 1, colored red (Contaminants and Risk Assessment), focuses on emerging contaminants, pharmaceuticals, and water risks, while Cluster 2, colored green (Environmental Analysis and Toxicity), highlights environmental analysis and toxicity. This targeted approach underlines the importance of understanding the fate and transport of pharmaceuticals, assessing their potential risks, and developing effective strategies to mitigate their environmental impact, including the use of monitoring-based studies to track the presence and levels of pharmaceutical contamination in different environmental areas.

The VOSviewer network visualization presented in Figure 2 illustrates the interconnectedness of 32 keywords. As also observed in Figure 1, the keyword ‘pharmaceuticals’ stands out as the central theme, with strong connections to areas such as wastewater treatment, risk assessment, and environmental monitoring. This highlights the multidisciplinary nature of this field, which contains analytical techniques, remediation technologies, and the evaluation of environmental and health impacts. The network is organized into six clusters, each representing a specific research focus. Cluster 1 (red color), Treatment Processes, focuses on technologies for removing pharmaceuticals from water, including adsorption, advanced oxidation processes, and wastewater treatment. Cluster 2 (green color), Environmental Risks and Impacts, addresses the presence and risks of pharmaceuticals in various environments, including emerging contaminants, pollution, and risk assessment. Cluster 3 (dark blue color), Analytical Techniques and Environmental Matrices, centers on analytical methods, including UHPLC-MS/MS; extraction process (solid-phase extraction, SPE); and various water matrices. Cluster 4 (mustard color), Environmental Monitoring and Impact, highlights the monitoring and assessment of pharmaceutical contamination and its environmental impacts. Cluster 5 (purple color), Toxicity and Water Pollution, focuses on the toxicological effects of pharmaceuticals and their contribution to water pollution. Cluster 6 (light blue color), Environmental Context, highlights the broader environmental aspects related to pharmaceuticals in water, including treatment processes and their impacts on the environment.

Important subjects, including pollutant detection, monitoring campaigns, risk assessment, and treatment strategies, are highlighted in Figure 1 and Figure 2. The importance of the keywords ‘environmental monitoring’, ‘detection’, and ‘occurrence’ in these visualizations underscores the crucial role of monitoring studies in understanding the fate and transport of pharmaceuticals in the environment. These studies are essential for the tracking of pharmaceuticals, analyzing their behaviors, and assessing potential ecological and health risks. Moreover, monitoring studies provide data crucial for evaluating the quality of water bodies. The VOSviewer analysis performed outlines the contribution of the present study to the enhancement of this knowledge and the filling of the existing research gaps.

The analysis of a high number of pharmaceutical compounds in different environmental matrices provides a more comprehensive understanding of pharmaceutical pollution, in comparison to previous studies, capturing a wider range of potential contaminants. To assess the ecological implications of the concentrations detected, this study employed Risk Quotient (RQ) analysis, evaluating potential risks to different trophic levels. By conducting seasonal sampling campaigns, this research also highlights temporal variations in contamination levels. The influence of WWTP discharges on freshwater systems was evaluated, emphasizing the need for advanced treatment strategies to mitigate the observed risks. This research highlights the importance of monitoring pharmaceutical pollution, aiming to raise awareness about environmental risks and the need for research on quaternary treatments.

Performing monitoring studies of pharmaceutical compounds necessitates a deep involvement and collaboration between the scientific community and the supervisors of the WWTPs. These partnerships are considered a key factor in reaching the goals of the new Urban Wastewater Directive (EU) 2024/3019 [17], which concerns the identification of target compounds and the definition of treatment achievements. The study also contributes by providing new monitoring data, which fulfills objectives of the European Commission’s fifth Watch List [18].

## 2. Materials and Methods

### 2.1. Sampling Campaign

**Figure 3:**
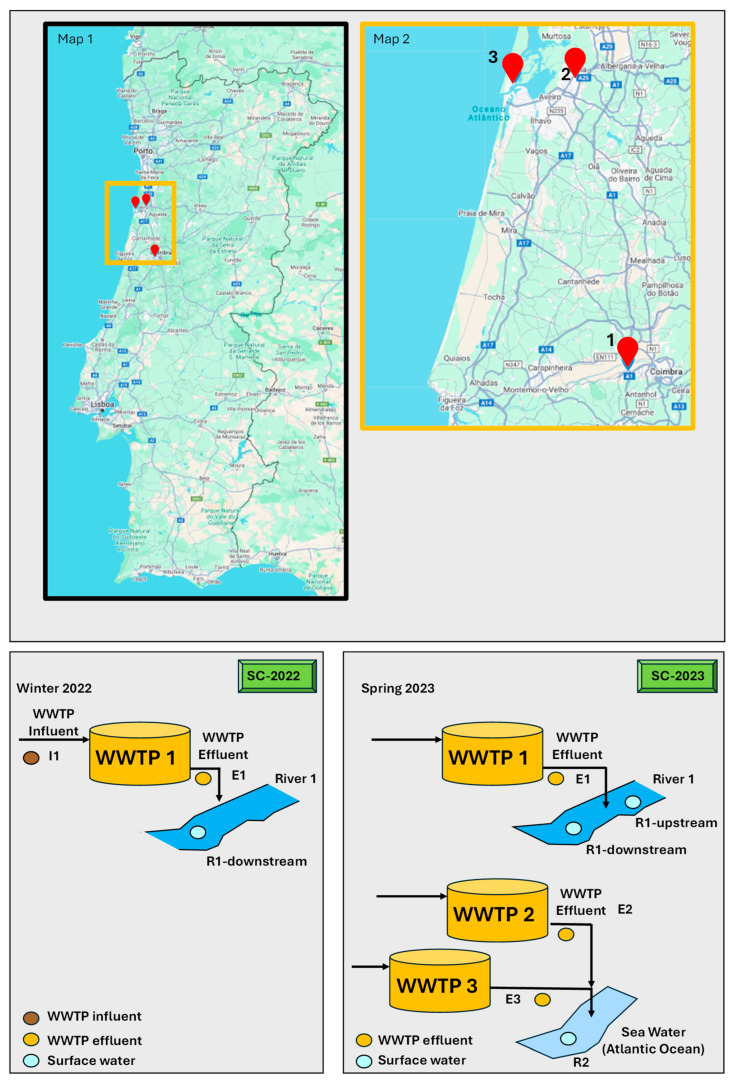
(**Top**) Map of the study area, showing Portugal (Map 1) and the regional locations of the wastewater treatment plants (Map 2). (**Bottom**) Schematic diagram of the sampling sites of the campaigns conducted in 2022 (SC-2022, winter sampling of WWTP 1 and receiving water R1) and 2023 (SC-2023, spring sampling of WWTPs 1, 2, and 3, and receiving waters R1 and R2).

Two sampling campaigns were performed, and three WWTPs located in the central region of Portugal were the main focus (Figure 3). The first campaign, conducted in winter 2022, focused on WWTP 1’s influent and effluent, as well as its receiving water (River 1) downstream of the effluent discharge, to evaluate treatment performance and pollutant levels. The second campaign (in spring 2023) expanded the study, including effluents from WWTPs 1, 2, and 3. This broader approach allowed for a more comprehensive assessment of the impacts of the effluents on surface water quality, with a detailed analysis of River 1, both upstream (R1-upstream) and downstream (R1-downstream) of the effluent discharge. The receiving water (R2) of the effluents from WWTPs 2 and 3 is sea water, which was also sampled downstream of the discharge of the submarine outfall. The objectives of the present study were to assess wastewater treatment efficiency and seasonal variation, as well as their impacts on the receiving bodies of water.

### 2.2. Description of the Study WWTPs

WWTP 1 and WWTP 2 employ preliminary treatment followed by primary sedimentation and secondary treatment using the activated sludge (extended aeration) process. WWTP 3 utilizes preliminary treatment followed by primary sedimentation and secondary treatment by aerated lagoon. The locations of the three WWTPs on the map of Portugal (Maps 1 and 2), along with a schematic drawing of the sampling campaigns (SC-1 and SC-2), are illustrated in Figure 3. WWTP 1 serves approximately 45,000 inhabitants, with an average daily flow of 1700 m^3^ and discharge into River 1. WWTP 2, the largest plant, serves approximately 272,000 inhabitants, with an average daily flow of 48,705 m^3^, while WWTP 3 serves approximately 9400 inhabitants, with an average daily flow of 1065 m^3^. Both WWTP 2 and WWTP 3 discharge through a submarine outfall. WWTP 1 is 55 km from WWTP 2 and 55 km from WWTP 3, while WWTP 2 is 11 km from WWTP 3.

### 2.3. Collection and Pre-Treatment of the Samples

High-density polyethylene bottles were rinsed with ultra-pure water and then with the sampled waters before the collection of samples. Surface water samples were collected as grab samples (1 L), composed of a single aliquot taken at a specific site and exact time to reflect the instantaneous water characteristics. Wastewater samples were collected as 24 h composite samples (1 L), composed of multiple aliquots obtained over time to represent average wastewater characteristics. In each sampling campaign, samples were transported in an ice-cooled hermetic box. Upon arrival at the laboratory, samples were vacuum-filtered through a 0.45 µm nylon membrane filter (FilterBio, Nantong, China) and immediately extracted using SPE before UHPLC-MS/MS analysis (Shimadzy, Tokyo, Japan).

### 2.4. Eluents, Solvents, and Reagents

The eluents used in the UHPLC-MS/MS analysis included ultra-pure water, methanol, acetonitrile, and propanol, all of MS grade. Additional reagents, such as the formic acid, hydrochloric acid, and sodium hydroxide used for pH adjustment and the ethylenediaminetetraacetic acid disodium salt dihydrate (Na_2_EDTA) used as a chelating agent, were also employed. For the conditioning, equilibration, and elution steps in the SPE extraction, ultra-pure water and methanol of appropriate purity grades were used. Detailed specifications, including supplier information and specifications, are provided in Table S1 of the Supplementary Materials.

### 2.5. Targeted Pharmaceutical Compounds

This study targeted 97 pharmaceuticals from various therapeutic classes, including analgesics, nonsteroidal anti-inflammatory drugs (NSAIDs), antibiotics, psychiatric drugs, cardiovascular drugs (β-blockers, statins, fibrates, and calcium channel blockers), gastrointestinal drugs (proton pump inhibitors, laxatives), antidiabetic drugs, stimulants, anorexics, and pharmaceuticals used in the treatment of Alzheimer’s and Parkinson’s diseases. Table S2 in the Supplementary Materials presents details on the pharmaceuticals, their metabolites and degradation products, isotopically labeled internal standards (ILIS), CAS numbers, molecular formulas, molecular weights, and supplier companies, as well as the solvents used to prepare the stock solutions.

Stock standards for each analyte were prepared at 1 g/L and stored at −20 °C. Working standard solutions containing all compounds were prepared in acetonitrile:ultra-pure water (30:70, *v*/*v*). A mixture of ILIS was prepared for internal standard calibration.

### 2.6. Analytical Method

#### 2.6.1. Sample Pre-Treatment

The SPE used for extracting the target compounds from the collected samples is based on approaches described in previous authors’ work [8,18]. A 0.1 M Na_2_EDTA solution was added to each type of sample (50 mL of WWTP influent, 100 mL of WWTP effluent or 250 mL of surface water) to obtain a final concentration of 0.1%. Afterward, the sample pH was adjusted to pH 2 using HCl. The pre-treated samples were passed through Strata-X SPE cartridges (Phenomenex, Inc., Torrance, CA, USA) conditioned with 5 mL of methanol and equilibrated with 5 mL of ultra-pure water followed by 5 mL of ultra-pure water at pH 2. After loading the sample, 5 mL of ultra-pure water was passed through the column. Then, the cartridges were dried under a continuous vacuum for 1 h. Analytes were eluted with methanol, evaporated, and reconstituted with acetonitrile:water (30:70, *v*/*v*). For each sample, two extractions were performed.

A 5 µL ILIS mixture was added to both standards and samples, resulting in the following final concentrations: 800 µg/L for acetaminophen-d4, 900 µg/L for azithromycin-d3, 100 µg/L for carbamazepine-d10, 400 µg/L for caffeine ^13^C_3_, 200 µg/L for diazepam-d5, 200 µg/L for dl-methamphetamine-d5 hydrochloride, 60 µg/L for fluoxetine-d5 hydrochloride, 250 µg/L for gemfibrozil-d6, 1000 µg/L for ibuprofen-d3, 50 µg/L for metformin-(dimethyl-d6) hydrochloride, 55 µg/L for salicylic acid-d4, 400 µg/L for sulfamethoxazole-d4, 150 µg/L for topiramate-d12, and 100 µg/L for venlafaxine-d6.

#### 2.6.2. Chromatographic Analysis

A UHPLC-MS/MS system (Shimadzu Corporation, Kyoto, Japan) with two solvent delivery modules (LC-40AD xs), a degasser (DGU-20A5), an autosampler (SIL-30AC), a column oven (CTO-20A), and a system controller (CBM-20A) coupled to a triple-quadrupole mass spectrometer, operating in electrospray ionization (ESI) mode, was used to analyze the 97 pharmaceuticals. The quantification of the analytes was performed using Multiple Reaction Monitoring (MRM). Lab Solutions software (Shimadzu Corporation, Kyoto, Japan), version 5.80, was used for system control and data processing. Argon was used as the collision-induced dissociation gas (CID) at a pressure of 230 kPa, and nitrogen was used as the nebulizing and drying gas. The mass spectrometry conditions are detailed in the authors’ previous works [7,19] and presented in Table S3 (Supplementary Materials).

Six chromatographic programs were used for the compounds analyzed in the UHPLC-MS/MS equipment (Shimadzu, Kyoto, Japan). All programs maintain an oven temperature of 30 °C and a 5 µL injection volume. The chromatographic programs vary as to % acetonitrile at the beginning of the chromatographic program, ramp rates, and hold times. Chromatographic conditions, eluents, modes of elution, and mass spectrometry source-dependent parameters used in each chromatographic program are presented in Table S4 (Supplementary Materials).

### 2.7. Analytical Method Validation

Linearity, method detection limits (MDLs), method quantification limits (MQLs), precision (intra- and inter-day), recovery, and matrix effect (ME) were evaluated for validation purposes.

### 2.8. UHPLC-MS/MS Analytical Sequence

Following sample preparation and extraction, the resulting extracts were analyzed using UHPLC-MS/MS, following a structured injection sequence to ensure data quality and analytical reliability. The analytical run began with three or more injections of solvent (initial mobile phase composition) to verify baseline stability. This was followed by triplicate injections of calibration standards to construct the calibration curve. To assess system precision, two standards (at 100 µg/L and 1000 µg/L) were each injected six times. After, to confirm the absence of carryover, an additional triplicate solvent injection was performed. Each sample extract was then injected in triplicate, with solvent injections placed between different sample extracts to continuously monitor for carryover. At the end of the batch run, the same two standards (100 µg/L and 1000 µg/L) were re-injected six times to verify system stability and reproducibility. Several additional solvent injections were also performed at the end to ensure cleanliness and a stable baseline.

For ME and recovery tests, the injection sequence starts with the injection of the solvent (mobile phase composition), followed by triplicate injections of standards prepared in solvent, then triplicate injections of standards prepared in matrix (post-spiked samples), and finally, triplicate injection of the pre-spiked SPE sample extracts (samples that were contaminated with a known concentration before SPE extraction). At the end of the sequence, several injections of the solvent are performed.

### 2.9. Environmental Risk Characterization

The potential risks to the aquatic environment posed by the detected pharmaceuticals in the studied surface waters were assessed using the RQ, calculated according to EU guidelines [20]. Predicted EC_50_ or LC_50_ values for each compound were obtained using the ECOSAR (Ecological Structure Activity Relationships) predictive model (v1.11) [21] across three key trophic levels: green algae, daphnids, and fish. These organisms were selected because they represent ecologically relevant and sensitive groups within the aquatic food web—algae as primary producers, daphnids as representative invertebrate primary consumers, and fish as higher-level vertebrate consumers. Their inclusion provides a broad perspective on potential ecological impacts and aligns with regulatory frameworks, making these levels suitable for standardized and comparable risk assessments [22]. RQ values were calculated using the highest measured concentration of each analyte to reflect a worst-case exposure scenario. For analytes detected below the method detection or quantification limits (MDLs or MQLs), half the MQL value was used as a conservative estimate [6]. An RQ ≥ 1 was interpreted as indicating potential environmental risk, while values < 1 were considered to represent negligible risk [23].

## 3. Results and Discussion

### 3.1. Validation Results

The linearity of the method was evaluated by constructing calibration curves using linear regression analysis. These curves were generated with eleven concentration points ranging from 1.0 to 1000 μg/L, obtaining determination coefficients of ≥0.9980. For all studied compounds, the good linearity of the method across the tested concentration ranges indicates its suitability for accurate quantitative analysis.

Pharmaceutical recovery was assessed in surface water and wastewater samples. Recovery percentages were calculated by comparing the quantification ion areas of pre-spiked and post-spiked samples when SPE extraction was performed. Recovery tests were conducted using five surface water samples and three wastewater samples. Each sample was fortified and extracted in triplicate, and the average recovery for each pharmaceutical was calculated per sample type. The recovery percentages were then grouped into four ranges (>75%, >50–<75%, >25–<50%, and <25%) and are presented as pie charts in Figure S1 (Supplementary Materials).

Recovery distribution in surface water samples was similar for the two higher percentage ranges. Specifically, 42.3% of recovered pharmaceuticals were in the >75% recovery category, and 41.2% were in the >50–75% recovery range, indicating a robust overall recovery. Lower recovery percentages were observed, with 6.2% in the >25–50% recovery range and 10.3% below 25% recovery. For wastewater samples, the majority (75.3%) fell within the >75% category. The >50–75%, >25–50%, and <25% categories had 4.1%, 11.3%, and 9.3%, respectively.

According to USGS Method No. 5–B10, acceptable recovery ranges for analytical methods applied to environmental water samples typically lie between 60 and 130% [24]. The recovery values obtained in our research align with these standards, reinforcing the validity and precision of our analytical approach. Additionally, U.S. EPA Method 1694 [25] acknowledges that recovery rates can vary significantly—some compounds may exhibit high recoveries, while others might yield values below 50%. Furthermore, Almeida [26] noted that although recoveries exceeding 70% are generally more favorable, lower values, even those under 40%, can still be deemed acceptable if they show consistent reproducibility, thus enabling meaningful detection of pharmaceuticals in environmental water samples.

Most pharmaceuticals in surface water and wastewater samples had higher recoveries. Sample adjustment to pH 2 enhances analyte interaction with the Strata-X adsorbent. Lanzoprazole in surface water, and erythromycin, synephrine, metformin, benserazide, carbidopa, and pramipexole in both surface water and wastewater, had recovery determinations lower than 10%. At pH 2, these pharmaceuticals are not effectively retained in the Strata-X cartridge.

**Figure 4:**
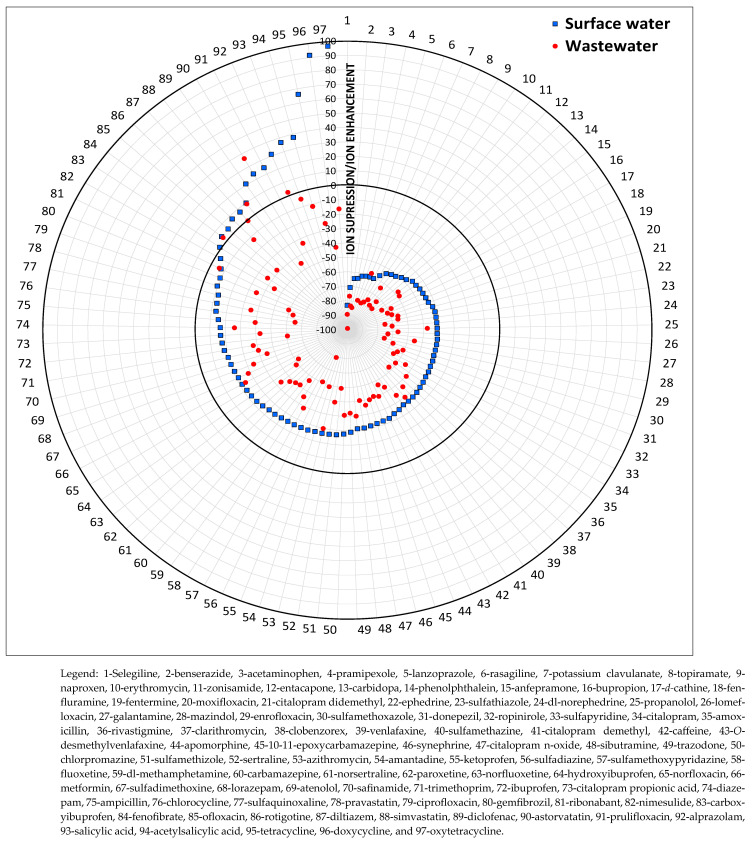
Matrix effect for 97 pharmaceuticals in surface water and wastewater samples.

The ME was evaluated for each compound by comparing the peak area of a standard prepared in solvent (acetonitrile:ultra-pure water, 30:70, *v*/*v*) with the area of the standard prepared in the matrix at 500 μg/L. Since water samples can contain the target compounds, blanks (non-spiked samples) were also analyzed, and the levels found were subtracted from those obtained for spiked samples. The absence of ME, ion suppression, and ion enhancement could be observed. ME values between ±20% indicate minimal ME. The results obtained are shown in the radar graph in Figure 4.

Ion suppression was the predominant ME in surface water (82 compounds) and wastewater (93 compounds) samples. Notably, the 82 compounds that exhibited suppression in surface water were the same compounds exhibiting ion suppression in wastewater. However, the remaining 15 compounds varied between ion suppression and ion enhancement, depending on the matrix. The graphical analysis (Figure 4) also highlights that ion suppression is more intense in wastewater, indicating a higher concentration of interfering components. This is supported by the average ME values, which were higher in wastewater (−50.3%), compared to the average ME for surface water (−24.5%). These findings align with previous studies reporting stronger matrix effects in wastewater than in other water types, along with lower matrix complexity [27,28,29,30].

For precision tests, two concentrations were tested (100 and 1000 µg/L) for all compounds, and the maximum RSD of intra- and inter-day analyses was lower than 10%.

MDLs and MQLs were also determined, and signal-to-noise ratios of 3 and 10 were used. When the target compound was detected in the samples, those samples were used for limit determination; otherwise, spiked samples were used. The results obtained are presented in Table S5 (Supplementary Materials).

Table S5 (Supplementary Materials) presents a wide variation in MDLs across the studied pharmaceuticals, with wastewater MDLs significantly higher than those in surface water. The variation in MDLs observed between surface water and wastewater samples can be attributed to the higher complexity of the wastewater matrix. Wastewater typically contains organic matter and various interfering substances, all of which can affect the sensitivity of the analytical method and increase the detection and quantification limits, compared to surface water [31]. Some compounds have very low detection limits, indicating they can be detected at trace concentrations, while others have considerably higher MDLs. The highest MDL values were those determined for norsertraline, oxytetracycline, atenolol, chlorocycline, and propranolol; of these, propranolol was associated with the highest MDL value (57.2 ng/L for surface water and 182 ng/L for wastewater).

### 3.2. Determination of Pharmaceutical Compounds

Table S6 (Supplementary Materials) presents the analytical data obtained from the studied compounds’ detections and concentrations, based on two sampling campaigns conducted in different seasons. These campaigns targeted wastewaters (WWTP effluents and influents) and surface waters that receive the effluents from the studied WWTP. The objective of the present study was to provide a temporal analysis and matrix assessment of pharmaceutical contamination. The discussion focuses on the (i) detected pharmaceuticals, as to quantity, concentration, and frequency; (ii) comparison of sampling campaigns performed in 2022 and 2023; (iii) highest concentration levels of the detected compounds; (iv) comparison of R1-upstream vs. R1-downstream in the 2023 sampling campaign; (v) comparison of influent I1 vs. effluent E1 in the 2022 sampling campaign; (vi) analyses of pharmaceutical detection across therapeutic classes; (vii) comparisons of pharmaceuticals and their transformation products; and (viii) risk assessment

#### 3.2.1. Detected Pharmaceuticals: Quantity, Concentration, and Frequency

**Figure 5:**
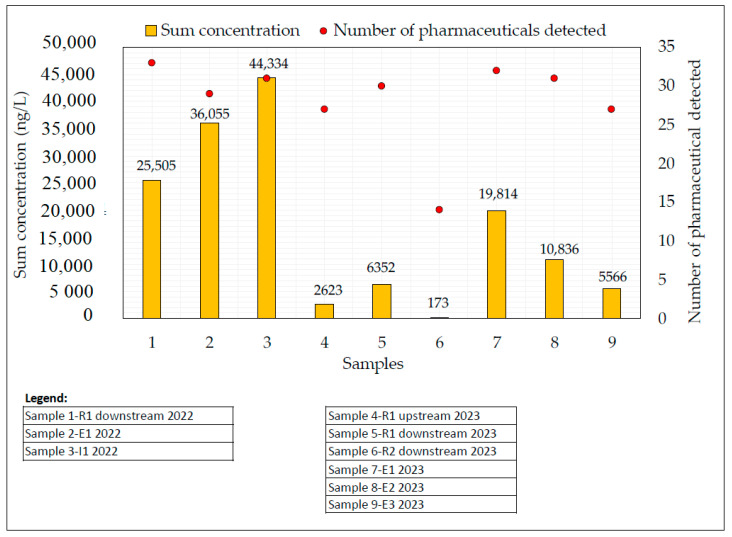
The sum of concentration determinations and the number of pharmaceuticals detected in both sampling campaigns, considering all studied samples.

The main results obtained are presented in Table S6 (Supplementary Materials) and shown in Figure 5.

The following discussion will be focused on several key topics: detection summary and overview, spatial distribution of pharmaceutical detections, influence of WWTP characteristics on detection patterns, temporal variations in wastewater samples, trends in pharmaceutical concentrations, exceptions to expected dilution patterns, potential sources of elevated downstream concentrations, and pharmaceutical detection frequency and the environmental implications of these determinations.

##### Detection Summary and Overview

Analyses of the surface water and WWTP wastewater samples (Table S6, Supplementary Materials and Figure 5) show that 37 compounds were found in at least one surface water sample, 36 in at least one wastewater sample, and 39 (40.2% of the analyzed compounds) in at least one sample, across all types. Notably, 12 compounds were consistently detected in surface water, while 24 compounds were found in all wastewater samples. 

##### Spatial Distribution of Pharmaceutical Detections

To visualize the distribution of pharmaceutical detections, Figure 5 presents the number of detected pharmaceuticals and the sum of concentrations (ng/L) for each sample collected.

Surface water R1, directly influenced by the effluent discharge from WWTP 1, presents the highest number of detections, with 33 compounds in the 2022 campaign and 27 (upstream) and 30 (downstream) in the 2023 campaign. In contrast, sampling point R2 (2023), which receives effluents from WWTP 2 and WWTP 3, showed the lowest number of detections, with 14 compounds.

With respect to the wastewater samples, the sampling campaign performed in 2023 had the highest and the lowest pharmaceutical detections, respectively, with 32 compounds in E1 and 27 compounds in E3. Surface water and wastewater results demonstrate the interactions between wastewater effluent discharge, dilution into surface water, and downstream pharmaceutical distribution (Figure 5).

##### Influence of WWTP Characteristics on Detection Patterns

The observed variations in pharmaceutical detections can be attributed mainly to differences in the dimensions and characteristics of the population agglomerate served by each WWTP. Although there are differences in the characteristics of the biological treatments, the treatment performance is similar, given the need to respect the legal requirements. The studied WWTPs varied significantly in size (Section 2.2), with WWTP 2 being the largest (approximately 272,000 inhabitants), followed by WWTP 1 (45,000 inhabitants) and WWTP 3 (9400 inhabitants). Although WWTP 1 is not the largest WWTP, the highest effluent concentrations are observed there, which can be related to the characteristics of the raw wastewater. In this case, it receives contributions from a hospital.

Surface water R2 was sampled downstream of the discharge of the submarine outfall of WWTPs 2 and 3 (Figure 3). Considering the large size of WWTP 2, it was expected that a higher number of detections and higher concentrations of the pharmaceutical compounds would be observed (Table S6, in the Supplementary Materials). However, a reduced number of compounds was detected at this sampling point, and lower concentrations were measured, due to the effect of dilution into the seawater (Figure 5).

##### Trends in Pharmaceutical Concentrations

The sums of the concentrations varied significantly, depending on the sample type and sampling campaign (Figure 5). The highest sums of the concentrations in wastewater and surface water samples were observed for the samples collected during the winter season (2022), with 44,334 ng/L in I1, 36,055 ng/L in E1, and 25,505 ng/L in R1. The highest sum of concentrations observed in I1 indicates a significant initial load of pharmaceuticals entering the wastewater treatment plant. The lowest value was observed in surface water R2 in 2023, with 173 ng/L. Significantly higher concentrations were observed in wastewater samples (E1, E2, and E3), compared to the receiving surface waters in both sampling campaigns (Table S6, Supplementary Materials), aligning with findings from other researchers [5,6,7,19,32,33] that similarly reported high concentrations in WWTP effluents, compared to receiving surface water.

##### Exceptions to Expected Dilution Patterns

Upon discharge into the aquatic environment, effluents undergo dilution due to the larger receiving water volume, which leads to a reduction in pollutant concentrations [34]. However, exceptions to the general profile were observed. In the 2022 sampling campaign, some compounds showed higher concentrations in R1-downstream compared to E1, including atorvastatin (848 ng/L in E1, 1620 ng/L in R1-downstream), citalopram propionic acid (157 ng/L in E1, 311 ng/L in R1-downstream), ciprofloxacin (n.d. in E1, 152 ng/L in R1-downstream), *O*-desmethylvenlafaxine (2011 ng/L in E1, 2304 ng/L in R1-downstream), gemfibrozil (15.2 ng/L in E1, 19.2 ng/L in R1-downstream), ketoprofen (1247 ng/L in E1, 1338 ng/L in R1-downstream), topiramate (238 ng/L in E1, 392 ng/L in R1-downstream), zonisamide (n.d. in E1, 34.4 ng/L in R1-downstream), and amantadine (n.d. in E1, 233 ng/L in R1-downstream). Similarly, in the 2023 sampling campaign, bupropion (below MDL in E1, 22.6 ng/L in R1-downstream) and metformin (92.0 ng/L in E1, 108 ng/L in R1-downstream) also had higher concentrations in R1-downstream when compared with the sample collected in E1.

##### Potential Sources of Elevated Downstream Concentrations

Although WWTP effluents are generally expected to have higher pharmaceutical concentrations than found in the receiving waters, several factors can lead to the opposite trend. Pre-existing contamination from upstream sources, runoff, or unknown pollution inputs may contribute to increased concentrations in the river [5,6,7]. Additionally, pollutants accumulated in sediments or biota can be released, increasing environmental loads beyond those in WWTP effluent discharges, even when input levels are relatively low [35]. Similar findings have been reported in monitoring studies conducted by the authors from 2013 to the present [5,6,7,36,37], suggesting the presence of unknown sources of contamination near these sampling points.

##### Frequency of Pharmaceutical Detection and Relevant Environmental Implications

A total of twelve compounds (acetaminophen, ampicillin, azithromycin, caffeine, fluoxetine, gemfibrozil, 2-hydroxyibuprofen, ibuprofen, ketoprofen, mazindol, naproxen, and salicylic acid) were detected at 100% frequency in both surface water and wastewater samples, indicating widespread and persistent environmental occurrence. Furthermore, an additional twelve compounds were detected at 100% frequency exclusively in the wastewater samples. These include alprazolam, atorvastatin, bupropion, carbamazepine, citalopram, clarithromycin, *O*-desmethylvenlafaxine, diclofenac, ofloxacin, sertraline, venlafaxine, and trazodone. These findings point to continuous input from anthropogenic sources and limited removal during conventional wastewater treatment.

The observed high detection frequency of pharmaceuticals within the NSAIDs/analgesics (acetaminophen, ibuprofen, ketoprofen, and naproxen), antibiotics (ampicillin, azithromycin, clarithromycin, and ofloxacin), and psychiatric drug classes (fluoxetine, bupropion, carbamazepine, citalopram, sertraline, venlafaxine, and trazodone) is supported by the high consumption of these pharmaceuticals, which leads to the continuous release of these compounds into wastewaters and consequently into the environment due to their inefficient removal in WWTPs, posing a significant threat to aquatic ecosystem health. Notably, the stimulant caffeine, an indicator of anthropogenic influence, showed high detection frequency and elevated concentration at certain sites. The ibuprofen metabolite 2-hydroxyibuprofen, known for its persistence, and the salicylic acid (acetylsalicylic acid degradation product) reflect the transformations and persistence of these compounds in the environment. Their ubiquitous presence underscores the need for improved monitoring, regulation, and treatment technologies to protect aquatic ecosystems.

This concern is reflected in recent regulatory developments. For instance, fluoxetine has been listed on the European Commission’s fifth Watch List [18] as a priority substance for environmental monitoring. Moreover, several compounds frequently detected in this study—including carbamazepine, citalopram, clarithromycin, diclofenac, and venlafaxine—are classified under Group I of the indicator substances in the newly adopted Urban Wastewater Directive (EU) 2024/3019 [17]. These regulatory actions highlight the growing recognition of the ecological risks posed by pharmaceutical contaminants and the need for targeted policy measures.

##### High-Concentration Compounds and Their Environmental Significance

A set of these compounds was detected at high concentrations, exceeding the microgram per liter (µg/L) level in surface water or wastewater samples (Table S6, Supplementary Materials), suggesting a higher potential for environmental impact. The consistent detection of atorvastatin, caffeine, *O*-desmethylvenlafaxine, diclofenac, 2-hydroxyibuprofen, ibuprofen, and ketoprofen at these levels in both water matrices may indicate their significant environmental persistence and/or limitations in their removal. The presence of other compounds, such as acetaminophen, carbamazepine, carboxyibuprofen, salicylic acid, and venlafaxine, at µg/L levels, specifically in wastewater effluent, further emphasizes their discharge into aquatic environments. The presence of these compounds in both WWTP effluents and receiving waters raises concerns regarding environmental exposure and potential ecological impacts.

#### 3.2.2. Comparison of Sampling Campaigns Performed in 2022 and 2023

Samples E1 and R1, collected in 2022 (winter), were also sampled in 2023 (spring). Higher sums of concentrations were observed in 2022, when compared to 2023 for R1-downstream and E1, indicating potential seasonal variations in pharmaceutical consumption (Figure 5). Compared to the 2023 sampling, the sums of concentrations in E1 and R1 were 1.8 and 4.0 times higher during the winter 2022 sampling campaign. Vieno et al. [38] also observed seasonal differences in pharmaceutical concentrations, with 3 to 5 times higher ratios in winter, and in their study, it was reported that increased pharmaceutical consumption during cold seasons raises the environmental risk. While temporal trends in pharmaceutical concentrations were not observed in Southern California coastal waters, a location which is associated with relatively constant and warm year-round temperatures [39], Hedgespeth et al. [40] reported a higher probability of detecting acetaminophen in Charleston Harbor, South Carolina, during winter. This seasonal variation can be attributed to several factors, including reduced sunlight inhibiting photodegradation [38] and the increased pharmaceutical use in winter seasons for respiratory infections, pain, fever, and inflammation [41]. Future monitoring studies should be performed in the same WWTPs to complement this study and expand the period of the temporal analysis. 

#### 3.2.3. Highest Concentration Levels of the Detected Compounds

The highest detected concentrations (Table S6, Supplementary Materials) ranged from below MDL to 5623 ng/L for 2-hydroxyibuprofen (R1, 2022) in surface water and between <MDL to 12,664 ng/L for caffeine (WWTP E1, 2022) in wastewater samples. Among the detected compounds, the stimulant caffeine and the metabolites *O*-desmethylvenlafaxine, 2-hydroxyibuprofen, and salicylic acid exhibited the highest concentrations. Caffeine was detected at 7805 ng/L in E1 (2022), 12,664 ng/L in I1 (2022), 654 ng/L in R1 upstream (2023), 1226 ng/L in R1 downstream (2023), and 5703 ng/L in E1 (2023). *O*-desmethylvenlafaxine reached 2124 ng/L in E2 (2023) and 2364 ng/L in E3 (2023); 2-hydroxyibuprofen was found at 5623 ng/L in R1 (2022), while salicylic acid was detected at 107 ng/L in R2, respectively.

Santos et al. (2022) [42] identified caffeine as a key anthropogenic marker due to its widespread consumption in beverages and food products, as well as its use as a psychopharmaceutical stimulant; this is related to its frequent detection and high concentrations in environmental samples. Similarly, Rodrigues et al. [33] highlighted caffeine as one of the most frequently detected compounds in urban streams. These findings align with the results of several studies, including those by Luo et al. [43], Paíga et al. [5,7,19,37,44], Buerge et al. [45], Seiler et al. [46], and Nguyen et al. [47]. Furthermore, Li et al. [48] reported that caffeine was a dominant pharmaceutically active compound pollutant in freshwater systems.

*O*-desmethylvenlafaxine, 2-hydroxyibuprofen are metabolites, and salicylic acid a degradation product of widely consumed therapeutic classes (psychiatric drugs and NSAIDs). These families of pharmaceuticals are prevalent worldwide, contributing to their frequent detection in environmental waters. The compound 2-hydroxyibuprofen, identified as the primary environmental metabolite of ibuprofen, is more stable and exhibits lower removal rates compared to carboxyibuprofen. This leads to higher environmental concentrations, as observed in the study of Ferrando-Climent et al. [49]. The non-detection of acetylsalicylic acid can be attributed to its hydrolysis, which results in salicylic acid and acetic acid [50]. Similarly, the metabolite *O*-desmethylvenlafaxine, found at higher concentrations in our results, aligns with findings from Lajeunesse et al. [51] and Colzani et al. [52], who identified it as one of the most frequently detected compounds in their studies.

#### 3.2.4. R1-Upstream vs. R1-Downstream in the 2023 Sampling Campaign

The 2023 sampling campaign evaluated the impacts of effluent discharge from WWTP E1 on pharmaceutical concentrations in River R1 by comparing upstream and downstream samples. As expected, total pharmaceutical concentrations were higher downstream of the discharge point (Figure 5), confirming that WWTP E1 is a significant source of pollutants in River R1. Nonetheless, some exceptions to this profile were observed. Naproxen had a higher concentration upstream (277 ng/L) than downstream (196 ng/L), suggesting the possibility of the presence of an unidentified upstream source. Salicylic acid and 2-hydroxyibuprofen showed similar concentrations at the R1-upstream and R1-downstream sampling points, with 515 ng/L for 2-hydroxyibuprofen and approximately 100 ng/L for salicylic acid. For these three compounds, WWTP effluent E1 appeared to have no impact on the river downstream. However, analysis of the effluent sample confirmed their presence at 404 ng/L (naproxen), 1545 ng/L (2-hydroxyibuprofen), and 171 ng/L (salicylic acid), indicating their discharge into the study river by effluent E1 (Table S6, Supplementary Materials). These results suggest that the effluent did contribute to increases in the concentrations of these compounds. However, its impact at the downstream sampling location was masked by the dilution of these compounds along the river between the effluent discharge point and the R1-downstream sampling location. In contrast, other pharmaceuticals showed more pronounced effects relative to the effluent discharge, with ratios ranging from 1.38 (ibuprofen) to 7.62 (atorvastatin). Particularly high ratios were observed for atorvastatin (7.62), ketoprofen (6.22), carbamazepine (4.82), diclofenac (4.88), and venlafaxine (4.52). These high ratios indicate that the WWTP is a contributor to the increased levels of these compounds in the downstream river segment.

Similar trends have been reported in previous studies, with Aukidy et al. [32] and Domínguez-García et al. [53] documenting higher pharmaceutical concentrations downstream compared to upstream, indicating a consistent pattern.

#### 3.2.5. Influent I1 vs. Effluent E1 in the 2022 Sampling Campaign

In the 2022 sampling campaign, a comparative analysis between influent (I1) and effluent (E1) samples revealed that while the total number of detected pharmaceuticals remained relatively stable (31 in I1 and 29 in E1), certain compounds exhibited higher concentrations in the effluent than in the influent (Table 1). This unexpected observation indicates negative removal efficiencies for these compounds, suggesting that their concentrations increased during the treatment process.

Negative removal rates were observed for a range of pharmaceuticals, including alprazolam, atorvastatin, azithromycin, bupropion, carbamazepine, citalopram, clarithromycin, *O*-desmethylvenlafaxine, diclofenac, diltiazem, fluoxetine, gemfibrozil, 2-hydroxyibuprofen, ibuprofen, ketoprofen, mazindol, ofloxacin, sertraline, venlafaxine, trazodone, and topiramate. Such phenomena are often attributed to a few known factors: deconjugation of metabolites and desorption from sludge [54,55,56,57,58,59,60,61]. These mechanisms highlight the complexity of pharmaceutical behavior in WWTPs. Carbamazepine, citalopram, clarithromycine, diclofenac, and venlafaxine are pharmaceutical compounds included in category 1 of (EU) Directive 2024/3019 [17] for which it was desirable to reach an 80% removal efficiency.

Other compounds, such as caffeine (38% removal), citalopram propionic acid (20% removal), and naproxen (14% removal) exhibited low removal efficiencies, reflecting the diverse physicochemical properties and biodegradability of these substances.

Conversely, some pharmaceuticals demonstrated significant removal efficiencies achieved through the treatment process: acetaminophen (approximately 98% removal), ciprofloxacin (100% removal), carboxyibuprofen (approximately 86% removal), and salicylic acid (94% removal). Similar trends in pharmaceutical removal during wastewater treatment have been reported in other research studies. For instance, Radjenović et al. (2009) [62] observed near-complete removal of acetaminophen (99.9%), while compounds such as carbamazepine and gemfibrozil exhibited negligible elimination [62]. NSAIDs are known to undergo various transformation processes during treatment; however, their incomplete removal has been consistently documented in WWTP effluents [63,64,65,66]. Notably, diclofenac demonstrated no removal in our study, with higher concentrations in effluents than in influents, indicating potential breakdown of conjugates or desorption from sludge—a phenomenon also reported by Zorita et al. [66]. This compound has shown highly variable removal efficiencies in the literature, ranging from 0% to 90%, depending on the treatment configuration and operational conditions [59,67,68,69,70]. Additionally, negative removal rates have been documented for several psychiatric drugs, including fluoxetine, citalopram, sertraline, and venlafaxine, which may be attributed to deconjugation processes or release from solid phases, as observed by Golbaz et al. [71].

Regarding the sum concentration, the highest values were observed in the WWTP influent I1 (Figure 5), highlighting the effectiveness of the treatment process in reducing pharmaceutical loads. However, the significant levels in the effluent indicate that complete removal was not achieved, as described in Table 1 and mentioned in the previous paragraph.

These results highlight the urgent need to optimize existing treatment processes or implement advanced technologies, such as quaternary treatment steps, to enhance the removal efficiency with respect to persistent and ecotoxicologically relevant pharmaceuticals.

#### 3.2.6. Analysis of Pharmaceutical Detection Across Therapeutic Classes

**Figure 6:**
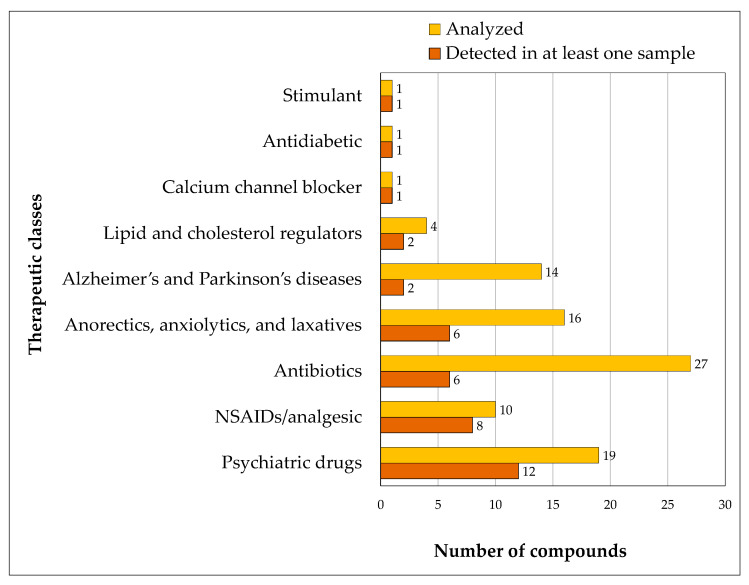
Compounds detected vs. as analyzed grouped by therapeutic classes.

A comparison of the number of compounds detected in at least one sample and the total number of compounds analyzed in each therapeutic class across all sample types is shown in the bar chart in Figure 6. Psychiatric drugs exhibited the highest number of detected compounds (12), indicating a broad presence of these substances in the analyzed samples. Following this class, NSAIDs/analgesics also had a substantial detection rate (8), suggesting that pain relievers and anti-inflammatory agents are also frequently encountered. Antibiotics, anorectics, anxiolytics, and laxatives showed moderate detection levels, with approximately six different pharmaceuticals identified in each category. Their high detection rates can be attributed to widespread use and potential environmental accumulation.

The increasing prevalence of mental health disorders and the long-term prescription of medications for conditions such as depression and anxiety contribute to the widespread consumption of these medications and their high levels of presence in water sources [72,73]. Similarly, the frequent over-the-counter availability of NSAIDs and analgesics results in high consumption rates, leading to their discharge into the aquatic environment [74,75]. Antibiotics also show a significant presence, reflecting their extensive use in both human and veterinary medicine [76,77]. In the cases of anxiolytics, anorectics, and laxatives, their detection in environmental samples suggests high consumption, often influenced by modern lifestyle habits, stress, and dietary concerns, raising potential ecological and public health concerns [78,79].

#### 3.2.7. Pharmaceuticals and Their Transformation Products

The detections and detected concentrations of parent compounds and their metabolites were compared across surface water and wastewater samples. Of the eleven targeted metabolites and degradation products, six were detected in at least one sample: carboxyibuprofen and 2-hydroxyibuprofen (metabolites of ibuprofen), salicylic acid (transformation product of acetylsalicylic acid), citalopram propionic acid (metabolite of citalopram), *O*-desmethylvenlafaxine (metabolite of venlafaxine), and 10,11-epoxycarbamazepine (metabolite of carbamazepine).

Table 2 presents the concentrations (ng/L) of the parent compounds, as well as those of their corresponding metabolites and degradation products, and the ratios between the metabolites and their respective parent compounds. Ratios higher than 1 illustrate the relative abundance and potential persistence of metabolites, as compared to their parent compounds, across wastewater and surface water samples.

Norsertraline was not detected; however, its parent compound, sertraline, was detected in 89% of the samples. The same profile was observed for norfluoxetine, which was not detected, although fluoxetine was detected in 100% of the samples. Carbamazepine was found at higher concentrations relative to its metabolite. Carbamazepine is extensively metabolized, with less than 2% of an oral dose excreted unchanged in urine [80]. Furthermore, Celiz et al. [81] reported that in biological treatments, some metabolites of carbamazepine can be partially retransformed into the parent compound through enzymatic and/or chemical transformation, potentially contributing to the persistence of the parent compound in the environment.

In contrast, the parent compound acetylsalicylic acid was not detected, but its degradation compound salicylic acid was detected in 100% of the samples. A similar profile was observed by Kosma et al. [82]; this phenomenon can be attributed to the hydrolysis of the parent compound into salicylic acid and acetic acid [50].

The same trend was observed for venlafaxine, in which *O*-desmethylvenlafaxine consistently exhibited higher concentrations than the parent compound. Magalhães et al. [83] indicated that venlafaxine is primarily excreted in human urine as metabolites (approximately 56% *O*-desmethylvenlafaxine, 16% *N,O*-didesmethylvenlafaxine, 1% *N*-desmethylvenlafaxine, and *N,O*-didesmethyl-N-desmethylvenlafaxine), with only a small fraction excreted as the unchanged compound (4.7%). This pattern is further supported by Schlüsener et al. [84], who observed that *O*-desmethylvenlafaxine concentrations were three times higher than those of venlafaxine. Similarly, the present study found *O*-desmethylvenlafaxine concentrations to be 2.9 to 4.4 times higher in surface water, 2.0 to 14 times higher in effluent WWTP samples, and 10 times higher in influent WWTP samples than the parent compound, respectively.

Regarding citalopram and its metabolites, citalopram propionic acid was only detected in the 2022 sampling campaign (I1, E1, and R1), in which its concentration was higher than that of its parent compound in samples I1 and R1. However, in the 2023 campaign, this metabolite was never detected, although the parent compound was detected.

The analysis of ibuprofen and its metabolites (2-hydroxyibuprofen and carboxyibuprofen) showed that 2-hydroxyibuprofen was the most persistent and frequently detected metabolite, with detections in six of the nine analyzed samples at the highest concentrations, as observed in the study of Ferrando-Climent et al. [49]. In contrast, ibuprofen had the highest concentration in two samples (R2 and E3) and carboxyibuprofen in one sample (influent I1).

#### 3.2.8. Risk Assessment

The environmental risk assessment based on RQ analysis identified several pharmaceuticals of potential ecological concern. Atorvastatin exceeded the risk threshold (RQ > 1) across all three trophic levels—fish (RQ = 1.27), daphnids (RQ = 8.57), and green algae (RQ = 15.7)—indicating broad ecological risk. Similarly, caffeine showed extremely high RQ values for algae (RQ = 214), followed by carbamazepine (RQ = 3.83) and venlafaxine (RQ = 1.22), suggesting significant risks to primary producers in aquatic ecosystems. The high RQ values for algae, which are primary producers, imply potential disruptions along the trophic chain, as algae form the foundation of the aquatic ecosystems. These findings underscore the need for monitoring pharmaceutical contamination, improving wastewater treatment technologies, and implementing regulatory measures to mitigate the risks associated with pharmaceutical discharge into aquatic environments. Although fluoxetine did not exceed the RQ > 1 threshold, its detection in all samples, and its known ecotoxicological effects, underscore the need for targeted monitoring and risk management.

The detection of carbamazepine and venlafaxine, both now included in Group I of the indicator substances under Directive (EU) 2024/3019 [17], further emphasizes the importance of enhancing removal performance in wastewater treatment plants. Removal of these substances is required to function at a minimum of 80% efficiency, reinforcing their regulatory and environmental relevance.

## 4. Conclusions

This study demonstrated significant pharmaceutical contamination in both surface waters and wastewaters, with 40.2% of the 97 monitored compounds being detected. Notably, seven compounds—including caffeine, diclofenac, and ibuprofen—were found at microgram-per-liter levels in both matrices, with caffeine reaching up to 12,664 ng/L in wastewater. Metabolites such as *O*-desmethylvenlafaxine and 2-hydroxyibuprofen were detected at higher concentrations than their parent compounds, suggesting high environmental persistence. The persistence and high concentrations of pharmaceuticals in aquatic environments, driven by their widespread use and resistance to degradation, pose significant ecological and human health concerns, highlighting the limitations of current wastewater treatment processes aiming to effectively remove them. The presence of fluoxetine in all samples—an active pharmaceutical included in the European Commission’s fifth Watch List—is of great concern, underscoring the need for targeted risk assessment and regulatory action. Also, carbamazepine, citalopram, clarithromycin, diclofenac, and venlafaxine, present in all WWTP samples, should be the foci of environmental policies in the future, as is already the case in the new Urban Wastewater Directive (UE) 2024/3019.

Seasonal trends were observed, with higher concentrations in winter, likely reflecting increased pharmaceutical consumption.

The ecological risk posed by the persistence of psychiatric drugs, NSAIDs, and antibiotics underlines the urgency of implementing stricter discharge regulations, public education campaigns on proper disposal, and continuous environmental monitoring. The alignment of these findings with the priorities of EU environmental policy frameworks underscores their broader relevance.

The environmental risk assessment identified several pharmaceuticals of notable ecological concern. Atorvastatin exceeded the risk threshold (RQ > 1) across all three trophic levels—fish, daphnids, and green algae. Caffeine, carbamazepine, and venlafaxine also presented high RQ values, particularly affecting algae (RQ = 214, 3.83, and 1.22, respectively). Although fluoxetine did not surpass the RQ > 1 threshold, its consistent detection across all samples and known ecotoxicity emphasize the need for targeted monitoring and regulatory attention. These findings reinforce the need for enhanced treatment technologies.

Given the significant and persistent contamination observed, there is a pressing need for improved wastewater treatment technologies (e.g., quaternary treatments), stricter regulations on pharmaceutical disposal, and enhanced public awareness. Also, continuous monitoring is essential for both a better understanding of the contamination patterns and mitigation of the potential risks posed by pharmaceutical compounds in aquatic environments. These objectives have been included recently in European regulations (e.g., the Urban Wastewater Directive (EU) 2024/3019 and the fifth EU Watch List), and it is desirable that new legal frameworks arise worldwide.

## Figures and Tables

**Table 1:** Concentration in ng/L of WWTP effluent and WWTP influent samples collected in the sampling campaign performed in 2022.

Pharmaceuticals	Concentration (ng/L)		Removal Efficiency (%)
	Wastewater 2022		
	WWTP 1	WWTP 1	
	Influent	Effluent	
Acetaminophen	3638	<MDL	98.4
Alprazolam	105	114	n.e.
Ampicillin	<MDL	<MDL	
Atorvastatin	236	848	n.e.
Azithromycin	121	634	n.e.
Bupropion	<MDL	62.4	n.e.
Caffeine	12,664	7805	38.4
Carbamazepine	177	1359	n.e.
Carboxyibuprofen	7068	998	85.9
Citalopram	34.3	218	n.e.
Citalopram propionic acid	196	157	19.9
Ciprofloxacin	59.2	n.d.	100
Clarithromycin	<MDL	15.2	n.e.
Clobenzorex	<MDL	n.d.	
O-desmethylvenlafaxine	639	2011	n.e.
Diclofenac	1164	4882	n.e.
Diltiazem	<MDL	34.0	n.e.
10,11-epoxy carbamazepine	<MDL	<MDL	
Fluoxetine	<MDL	34.4	n.e.
Gemfibrozil	13.3	15.2	n.e.
2-Hydroxyibuprofen	4215	7767	n.e.
Ibuprofen	3733	4442	n.e.
Ketoprofen	891	1247	n.e.
Mazindol	<MDL	12.4	n.e.
Naproxen	963	828	14.0
Ofloxacin	<MDL	167	n.e.
Salicylic acid	8353	468	94.4
Sertraline	<MDL	166	n.e.
Venlafaxine	64.2	1029	n.e.
Trazodone	<MDL	503	n.e.
Trimethoprim	<MDL	n.d.	
Topiramate	n.d.	238	n.e.

Abbreviation: n.e.—Not eliminated; n.d.—Not detected

**Table 2:** Concentrations (ng/L) of parent compounds and their corresponding metabolites or degradation products across wastewater and surface water samples, including the calculated concentration ratios of parent-to-metabolite and metabolite-to-parent.

Pharmaceutical, Metabolites, Degradation Products, and Associated Ratios	Concentration (ng/L)								
	Concentration (ng/L)					Concentration (ng/L)			
	2022	2022	2023	2023	2023	2022	2023	2023	2023
	I1	E1	E1	E2	E3	R1 Downstream	R1 Upstream	R1 Downstream	R2
Carbamazepine (PC)	177	1359	900	563	250	981	70.1	340	n.d.
10,11-epoxy carbamazepine (M1)	<MDL	<MDL	<MDL	<MDL	n.d.	<MDL	n.d.	<MDL	n.d.
Fluoxetine (PC)	<MDL	34.4	6.80	<MDL	<MDL	7.55	<MDL	<MDL	<MDL
Norfluoxetine (M1)	n.d.	n.d.	n.d.	n.d.	n.d.	n.d.	n.d.	n.d.	n.d.
Sertraline (PC)	<MDL	166	57.0	<MDL	<MDL	13.9	<MDL	20.7	n.d.
Norsertraline (M1)	n.d.	n.d.	n.d.	n.d.	n.d.	n.d.	n.d.	n.d.	n.d.
Acetylsalicylic acid (PC)	n.d.	n.d.	n.d.	n.d.	n.d.	n.d.	n.d.	n.d.	n.d.
Salicylic acid (DP)	8353	468	171	229	225	175	101	102	107
Ibuprofen (PC)	3733	4442	801	129	246	2267	176	242	28.6
Carboxyibuprofen (M1)	7068	998	n.d.	n.d.	n.d.	n.d.	38.9	n.d.	n.d.
2-Hydroxyibuprofen (M2)	4215	7767	1545	430	<MDL	5623	515	515	<MDL
*Ratio M1/PC*	1.89	0.22					0.22		
*Ratio M2/PC*	1.13	1.75	1.93	3.34		2.48	2.93	2.13	
Citalopram (PC)	34.3	218	140	142	31.2	162	18.9	54.4	n.d.
Citalopram demethyl (M1)	n.d.	n.d.	n.d.	n.d.	n.d.	n.d.	n.d.	n.d.	n.d.
Citalopram didemethyl (M2)	n.d.	n.d.	n.d.	n.d.	n.d.	n.d.	n.d.	n.d.	n.d.
Citalopram N-oxide (M3)	n.d.	n.d.	n.d.	n.d.	n.d.	n.d.	n.d.	n.d.	n.d.
Citalopram propionic acid (M4)	196	157	n.d.	n.d.	n.d.	311	n.d.	n.d.	n.d.
*Ratio M4/PC*	5.72	0.72				1.92			
Venlafaxine (PC)	64.2	1029	663	955	173	796	51.1	234	n.d.
*O*-Desmethylvenlafaxine (M1)	639	2011	1828	2124	2364	2304	225	745	n.d.
*Ratio M1/PC*	9.95	1.96	2.76	2.22	13.7	2.90	4.41	3.19	

Abbreviations: PC—Parent compound, M—Metabolite, DP—Degradation product, MDL—Method detection limit, n.d.—Not detected.

## Data Availability

Data are available upon reasonable request to the corresponding authors.

## Supplementary Materials

The following supporting information can be downloaded at: https://www.mdpi.com/article/10.3390/jox15030078/s1, Table S1: Technical specifications of eluents, reagents, and solvents used in the study, including supplier information and product details; Table S2: Pharmaceuticals, metabolites, degradation products, isotopically labelled internal standards (ILIS), chemical abstracts service (CAS), formula, molecular weight, supplier company, and solvent used for the preparation of each stock solution; Table S3: Therapeutic class, pharmaceuticals, ionization mode, precursor ions, product ions, mass spectrometry conditions, ion ratio, and isotopically labeled internal standards (ILIS) for each pharmaceutical in the study and chromatographic program (compounds were organized by alphabetic order in each chromatographic program); Table S4: Chromatographic conditions, eluents, mode of elution, and source-dependent parameters for each chromatographic program in the negative and positive ionization modes; Table S5: Method detection limits for each studied compound, for surface water and wastewater samples; Table S6: Concentrations (ng/L) of each studied compound in surface waters and WWTP wastewaters during the 2022 and 2023 sampling campaigns; Figure S1: Percentage of recovered pharmaceuticals within each recovery range, for surface water and wastewater.

## Abbreviations

The following abbreviations are used in this manuscript:

ECOSAR	Ecological Structure–Activity Relationships
ILIS	Isotopically Labeled Internal Standards
MDL	Method Detection Limit
ME	Matrix Effect
MQL	Method Quantification Limit
MRM	Multiple Reaction Monitoring
MS/MS	Tandem Mass Spectrometry
n.d.	Not detected
n.e.	Not eliminated
RQ	Risk Quotient
SPE	Solid Phase Extraction
UHPLC	Ultra-High-Performance Liquid Chromatography
