# Supplementary material for: Occurrence of 97 Pharmaceuticals in Wastewater and Receiving Waters: Analytical Validation and Treatment Influence
Source: J Xenobiot. 2025 May 23;15(3):78. doi: 10.3390/jox15030078 (PMC12193875; doi:10.3390/jox15030078)
Supplement: Supplementary file 1 [file jox-15-00078-s001.zip › jox-3602576-supplementary.pdf]

Supplementary Materials (SM)

for

Occurrence of 97 Pharmaceuticals in Wastewater and Receiving Waters: Analytical Validation and Treatment Influence

Paula Paíga[1], Sónia Figueiredo[1,*], Manuela Correia[1], Magda André[2], Roberto Barbosa[2], Sandra Jorge[2], Cristina Delerue-Matos[1]

[1] REQUIMTE/LAQV, Instituto Superior de Engenharia do Porto, Instituto Politécnico do Porto, Rua Dr. António Bernardino de Almeida, 431, 4249-015 Porto, Portugal; MC: mmb@isep.ipp.pt; CD-M: cmm@isep.ipp.pt

[2] Águas do Centro Litoral, SA, Grupo Águas de Portugal, ETA da Boavista, Avenida Dr. Luís Albuquerque, 3030-410 Coimbra, Portugal; MA: magda.almeida@adp.pt; RB: rbarbosa@adp.pt; SJ: s.jorge@adp.pt

* Correspondence: PP: pcbpa@isep.ipp.pt; SF: saf@isep.ipp.pt; Tel.: +351 228340500, fax: +351 228321159

Number of pages: 19

Number of tables: 6

Number of Figures: 1

List of tables

Tables		Page
Table S1	Technical specifications of eluents, reagents, and solvents used in the study, including supplier information and product details.	3
Table S2	Pharmaceuticals, metabolites, degradation products, isotopically labelled internal standards (ILIS), chemical abstracts service (CAS), formula, molecular weight, supplier company, and solvent used for the preparation of each stock solution.	4-7
Table S3	Therapeutic class, pharmaceuticals, ionization mode, precursor ions, product ions, mass spectrometry conditions, ion ratio, and isotopically labeled internal standards (ILIS) for each pharmaceutical in the study and chromatographic program (compounds were organized by alphabetic order in each chromatographic program).	8-12
Table S4	Chromatographic conditions, eluents, mode of elution, and source-dependent parameters for each chromatographic program in the negative and positive ionization modes.	13
Table S5	Method detection limits for each studied compound for surface water and wastewater samples.	14-16
Table S6	Concentrations (ng/L) of each studied compound in surface waters and WWTP wastewaters during the 2022 and 2023 sampling campaigns.	17-18

List of Figures

Figures		Page
Figure S1	Percentage of recovered pharmaceuticals within each recovery range for surface water and wastewater.	19

Table S1. Technical specifications of eluents, reagents, and solvents used in the study, including supplier information and product details.

**Eluents, reagents, and solvents used in the SPE extraction**		**Specifications**	**Supplier company (City, Country)**	
**Eluent**	Ultrapure water	HPLC LC-MS grade Hipersolv CHROMANORM®	VWR	(Gliwice, Poland, and Fontenay-sous-Bois, France)
	Methanol	LC-MS grade, assay ≥99.9%, Chromasolv™	Honeywell	(Seelze, Germany)
	Acetonitrile	LC-MS grade, assay ≥99.9%	Carlo Erba	(Val de Reuil Cedex, France)
	Propanol	MS grade	Sigma-Aldrich	(Steinheim, Germany)
**Reagents**	Formic acid	PA-ACS	Carlo Erba	(Rodano, Italy)
	Hydrochloric acid (HCl)	37%	Carlo Erba	(Rodano, Italy)
	Ethylenediaminetetraacetic acid disodium salt dihydrate (Na_2EDTA)		Panreac	(Barcelona, Spain)
	Sodium hydroxide (NaOH)		LabKem	(Barcelona, Spain)
**Solvents used in the SPE extraction (conditioning, equilibration, and elution)**	Ultrapure water	Ultrapure water was producure using a Milli-Q water purification system, Resistivity: 18.2 MΩ·cm	Millipore	(Molsheim, France)
	Methanol	CHROMASOLV™, gradient grade for HPLC, assay ≥99.9%	Honeywell	(Seelze, Germany)

Table S2. Pharmaceuticals, metabolites, degradation products, isotopically labelled internal standards (ILIS), chemical abstracts service (CAS), formula, molecular weight, supplier company, and solvent used for the preparation of each stock solution.

**Pharmaceuticals, metabolites, degradation products, and Isotopically Labelled Internal Standards** Note 1	**CAS** Note 2,3	**Formula (Molecular Weight)** Note 3	**Supplier Company**	**Solvent used for the preparation of each stock solution**
**Acetaminophen**	103-90-2	$C_8H_9NO_2$ (MW=151.17 g/mol)	Sigma-Aldrich (Madrid, Spain)	Acetonitrile
**Acetylsalicylic acid**	50-78-2	$C_9H_8O_4$ (MW=180.16 g/mol)	Sigma-Aldrich (Madrid, Spain)	Acetonitrile
**Alprazolam**	28981-97-7	$C_{17}H_{13}ClN_4$ (MW=308.77 g/mol)	Lipomed AG (Arlesheim, Switzerland)	Methanol
**Amantadine**	665-66-7	$C_{10}H_{17}N$ HCl (**Amantadine Hydrochloride**) (MW=187.71 g/mol)	Sigma-Aldrich (Madrid, Spain)	Ultrapure Water
	768-94-5	$C_{10}H_{17}N$ (**Amantadine**) (MW=151.25 g/mol)		
**Amfepramone**	134-80-5	$C_{13}H_{20}ClNO$ **(Amfepramone hydrochloride)** (MW=241.76 g/mol)	Lipomed AG (Arlesheim, Switzerland)	Methanol
	90-84-6	$C_{13}H_{19}NO$ (**Amfepramone**) (MW=205.30 g/mol)		
**Amoxicillin**	61336-70-7	$C_{16}H_{25}N_3O_8S$ (**Amoxicillin trihydrate)** (MW=419.45 g/mol)	Sigma-Aldrich (Madrid, Spain)	Methanol-Ultrapure water (2:1, v/v)
	26787-78-0	$C_{16}H_{19}N_3O_5S$ (**Amoxicillin**) (MW=365.40 g/mol)		
**Ampicillin**	7177-48-2	$C_{16}H_{19}N_3O_4S$ (MW=349.50 g/mol)	Sigma-Aldrich (Madrid, Spain)	Acetonitrile-metanol (1:1, v/v)
**Apomorphine**	41372-20-7	$C_{17}H_{17}NO_2$ HCl 1/2H_2O (**Apomorphine hydrochloride)** (MW=312.79 g/mol)	Sigma-Aldrich (Madrid, Spain)	Ultrapure Water
	58-00-4	$C_{17}H_{17}NO_2$ (**Apormorphine**) (MW=267.33 g/mol)		
**Atenolol**	29122-68-7	$C_{14}H_{22}N_2O_3$ (MW=266.34 g/mol)	Sigma-Aldrich (Madrid, Spain)	Methanol
**Atorvastatin**	344423-98-9<br>134523-00-5	$C_{66}H_{74}CaF_2N_4O_{13}$ (**Atorvastatin calcium trihydrate)** (MW=1209.41 g/mol)<br>$C_{33}H_{35}FN_2O_5$ (**Atorvastatin)** (MW=558.65 g/mol)	Sigma-Aldrich (Madrid, Spain)	Acetonitrile-5% acetic acid in Ultrapure water
**Azithromycin**	83905-01-5	$C_{38}H_{72}N_2O_{12}$ (MW=749.00 g/mol)	Sigma-Aldrich (Madrid, Spain)	Methanol
**Benserazide**	14919-77-8	$C_{10}H_{15}N_3O_5$ HCl (**Benserazide hydrochloride**) (MW=293.70 g/mol)	Sigma-Aldrich (Madrid, Spain)	Ultrapure Water
	322-35-0	$C_{10}H_{15}N_3O_5$ (**Benserazide**) (MW=257.25 g/mol)		
**Bupropion**	31677-93-7	$C_{13}H_{19}Cl_2NO$ (**Bupropion hydrochloride)** (MW=276.20 g/mol)	Lipomed AG (Arlesheim, Switzerland)	Methanol
	34911-55-2	$C_{13}H_{18}ClNO$ (**Bupropion)** (MW=239.74 g/mol)		
**Carbidopa**	38821-49-7	$C_{10}H_{14}N_2O_4$ H_2O (MW=244.24 g/mol)	Sigma-Aldrich (Madrid, Spain)	Methanol
**Caffeine**	58-08-2	$C_8H_{10}N_4O_2$ (MW=194.19 g/mol)	Sigma-Aldrich (Madrid, Spain)	Methanol
**Carbamazepine**	298-46-4	$C_{15}H_{12}N_2O$ (MW=236.27 g/mol)	Sigma-Aldrich (Madrid, Spain)	Methanol
**Carboxyibuprofen**	15935-54-3	$C_{13}H_{16}O_4$ (MW=236.27 g/mol)	Sigma-Aldrich (Madrid, Spain)	Acetonitrile
**Chlorpromazine**	69-09-0	$C_{17}H_{20}Cl_2N_2S$ (**Chlorpromazine hydrochloride)** (MW=355.32 g/mol)	Sigma-Aldrich (Madrid, Spain)	Methanol-Ultrapure water
	318.863	$C_{17}H_{19}ClN_2S$ (**Chlorpromazine)** (MW=318.86 g/mol)		
**Chlortetracycline**	64-72-2	$C_{22}H_{24}Cl_2N_2O_8$ (**Chlortetracycline hydrochloride)** (MW=515.34 g/mol)	Sigma-Aldrich (Madrid, Spain)	Acetonitrile-metanol (1:1, v/v)
	57-62-5	$C_{22}H_{23}ClN_2O_8$ (**Chlortetracycline)** (MW=478.88 g/mol)		
**Ciprofloxacin**	85721-33-1	$C_{17}H_{18}FN_3O_3$ (MW=331.347 g/mol)	Sigma-Aldrich (Madrid, Spain)	Ultrapure Water-10% acetic acid in ultrapure water (1:1, v/v)
**Citalopram**	59729-33-8	$C_{20}H_{21}FN_2O$ (MW=324.40 g/mol)	Sigma-Aldrich (Madrid, Spain)	Methanol
**Citalopram N-oxide**	62498-71-9	$C_{20}H_{22}ClFN_2O_2$ (**Citalopram N-oxide hydrochloride)** (MW=376.86 g/mol)	H. Lundbeck (Copenhagen, Denmark)	Methanol
	917482-45-2	$C_{20}H_{21}FN_2O_2$ (**Citalopram N-oxide)** (MW=340.40 g/mol)		
**Citalopram propionic acid***	**Not Available**	$C_{18}H_{14}FNO_3$ (MW=311.31 g/mol)	H. Lundbeck (Copenhagen, Denmark)	Methanol
**Clarithromycin**	81103-11-9	$C_{38}H_{69}NO_{13}$ (MW=747.96 g/mol)	Sigma-Aldrich (Madrid, Spain)	Methanol
**Clavulanate potassium**	61177-45-5	$C_8H_8NO_5K$ (MW=237.25 g/mol)	Sigma-Aldrich (Madrid, Spain)	Methanol-Ultrapure water (1:1,v/v)
**Clobenzorex**	5843-53-8	$C_{16}H_{19}Cl_2N$ (**Clobenzorex hydrochloride)** (MW=296.24 g/mol)	LGC (Middlesex, UK)	Methanol
	13364-32-4	$C_{16}H_{18}ClN$ (**Clobenzorex)** (MW=259.78 g/mol)		
***d*-Cathine**	2153-98-2	$C_9H_{14}ClNO$ (***d*-Cathine hydrochloride)** (MW=187.67 g/mol)	Lipomed AG (Arlesheim, Switzerland)	Methanol
	492-39-7	$C_9H_{14}ClNO$ (***d*-Cathine)** (MW=151.21 g/mol)		
**Demethylcitalopram**	97743-99-2	$C_{19}H_{20}ClFN_2O$ (**Demethylcitalopram hydrochloride)** (MW=346.83 g/mol)	H. Lundbeck (Copenhagen, Denmark)	Methanol
	62498-67-3	$C_{19}H_{19}FN_2O$(**Demethylcitalopram)** (MW=310.40 g/mol)		
**R(-)-Deprenyl hydrochloride (Selegiline hydrochloride)**	14611-52-0	$C_{13}H_{17}N$ HCl (**Selegiline hydrochloride**) (MW=223.74 g/mol)	Sigma-Aldrich (Madrid, Spain)	Ultrapure Water
	14611-51-9	$C_{13}H_{17}N$ HCl (**Selegiline**) MW=187.29 g/mol)		
**Diazepam**	439-14-5	$C_{16}H_{13}ClN_2O$ (MW=284.74 g/mol)	Lipomed AG (Arlesheim, Switzerland)	Methanol
**Diclofenac**	15307-79-6	$C_{14}H_{10}Cl_2NNaO_2$ (**Diclofenac sodium salt)** (MW=318.12 g/mol)	Sigma-Aldrich (Madrid, Spain)	Acetonitrile-metanol (1:1, v/v)
	15307-86-5	$C_{14}H_{11}Cl_2NO_2$ (**Diclofenac)** (MW=296.10 g/mol)		

(cont Table S2)

**Pharmaceuticals, metabolites, degradation product, and Isotopically Labelled Internal Standards**[Note 1]	**CAS** [Note 2,3]	**Formula (Molecular Weight)** [Note 3]	**Supplier Company**	**Solvent used for the preparation of each stock solution**
**Didemethylcitalopram**	1189694-81-2	$C_{18}H_{18}ClFN_2O$ (**Didemethylcitalopram hydrochloride)** (MW=332.80 g/mol)	H. Lundbeck (Copenhagen, Denmark)	Methanol
	62498-69-5	$C_{18}H_{17}FN_2O$ (**Didemethylcitalopram)** (MW=296.30 g/mol)		
**Diltiazem**	33286-22-5	$C_{22}H_{27}ClN_2O_4S$ (**Diltiazem hydrochloride)** (MW=450.98 g/mol)	Sigma-Aldrich (Madrid, Spain)	Methanol
	42399-41-7	$C_{22}H_{26}N_2O_4S$ (**Diltiazem)** (MW=414.50 g/mol)		
**Donepezil**	120011-70-3	$C_{24}H_{29}NO_3$ HCl (**Donepezil hydrochloride**) MW=415.95 g/mol)	Sigma-Aldrich (Madrid, Spain)	Ultrapure water
	120014-06-4	$C_{24}H_{29}NO_3$ (**Donepesil**) MW=370.50 g/mol)		
**Doxycycline**	24390-14-5	$C_{22}H_{24}N_2O_8$ HCl $0.5H_2O$ $0.5C_2H_6O$ (**Doxycycline hyclate)** (MW=512.94 g/mol)	Sigma-Aldrich (Madrid, Spain)	Acetonitrile-metanol (1:1, v/v)
	564-25-0	$C_{22}H_{24}N_2O_8$ (**Doxycycline)** (MW= 444.44 g/mol)		
**Enrofloxacin**	93106-60-6	$C_{19}H_{22}FN_3O_3$ (MW= 359.40 g/mol)	Sigma-Aldrich (Madrid, Spain)	Ultrapure Water-10% acetic acid in ultrapure water (1:1, v/v)
**Entacapone**	130929-57-6	$C_{14}H_{15}N_3O_5$ (MW= 305.29 g/mol)	Sigma-Aldrich (Madrid, Spain)	Ethanol
**(+)-Ephedrine**	134-71-4	$C_{10}H_{16}ClNO$ (**(+)-Ephedrine hydrochloride)** (MW= 201.69 g/mol)	Lipomed AG (Arlesheim, Switzerland)	Methanol
	134-72-5	$C_{10}H_{15}NO$ (**(+)-Ephedrine**) (MW=165.24 g/mol)		
**10,11-Epoxy carbamazepine**	36507-30-9	$C_{15}H_{12}N_2O_2$ (MW=252.27 g/mol)	Sigma-Aldrich (Madrid, Spain)	Methanol
**Erythromycin**	643-22-1	$C_{37}H_{67}NO_{13}$ (MW=733.94 g/mol)	Sigma-Aldrich (Madrid, Spain)	Methanol
**Fenfluramine**	404-82-0	$C_{12}H_{17}ClF_3N$ (**Fenfluramine hydrochloride)** (MW=267.72 g/mol)	LGC (Middlesex, UK)	Methanol
	404-82-0	$C_{12}H_{16}F_3N$ (**Fenfluramine**) (MW=231.26 g/mol)		
**Fenofibrate**	49562-28-9	$C_{20}H_{21}ClO_4$ (MW=360.83 g/mol)	Sigma-Aldrich (Madrid, Spain)	
**Fluoxetine**	56296-78-7	$C_{17}H_{19}ClF_3NO$ (**Fluoxetine hydrochloride**) (MW=345.79 g/mol)	Sigma-Aldrich (Madrid, Spain)	Methanol
	54910-89-3	$C_{17}H_{18}F_3NO$ (**Fluoxetine**) (MW=309.33 g/mol)		
**Galantamine**	1953-04-4	$C_{17}H_{21}NO_3$ HBr (**Galantamine hydrochloride**) MW=368.27 g/mol)	Sigma-Aldrich (Madrid, Spain)	Ultrapure water
	357-70-0	$C_{17}H_{21}NO_3$ HBr (**Galantamine**) MW=287.36 g/mol)		
**Gemfibrozil**	25812-30-0	$C_{15}H_{22}O_3$ (MW=250.34 g/mol)	Sigma-Aldrich (Madrid, Spain)	Methanol
**2-Hydroxyibuprofen**	51146-55-5	$C_{13}H_{18}O_3$ (MW=222.28 g/mol)	Sigma-Aldrich (Madrid, Spain)	Acetonitrile
**Ibuprofen**	15687-27-1	$C_{13}H_{18}O_2$ (MW=206.29 g/mol)	Sigma-Aldrich (Madrid, Spain)	Acetonitrile
**Ketoprofen**	22071-15-4	$C_{16}H_{14}O_3$ (MW=254.29 g/mol)	Sigma-Aldrich (Madrid, Spain)	Acetonitrile
**Lansoprazole**	103577-45-3	$C_{16}H_{14}F_3N_3O_2S$ (MW=369.36 g/mol)	Sigma-Aldrich (Madrid, Spain)	Methanol
**Lomefloxacin**	8079-52-8	$C_{17}H_{20}ClF_2N_3O_3$ (**Lomefloxacin hydrochloride**) (MW=387.81 g/mol)	Sigma-Aldrich (Madrid, Spain)	Ultrapure Water-10% acetic acid in ultrapure water (1:1, v/v)
	98079-51-7	$C_{17}H_{19}F_2N_3O_3$ (**Lomefloxacin**) (MW=351.35 g/mol)		
**Lorazepam**	846-49-1	$C_{15}H_{10}Cl_2N_2O_2$ (MW=321.16 g/mol)	Lipomed AG (Arlesheim, Switzerland)	Methanol
**Mazindol**	22232-71-9	$C_{16}H_{13}ClN_2O$ (MW=284.74 g/mol)	Lipomed AG (Arlesheim, Switzerland)	Methanol
**Metformin**	1115-70-4	$C_4H_{12}ClN_5$ (**Metformin hydrochloride**) (MW=165.63 g/mol)	Sigma-Aldrich (Madrid, Spain)	Methanol
	657-24-9	$C_4H_{11}N_5$ (**Metformin**) (MW=129.17 g/mol)		
**d,l-Methamphetamine**	300-42-5	$C_{10}H_{16}ClN$ (**d,l-Methamphetamine hydrochloride**) (MW=185.70 g/mol)	Lipomed AG (Arlesheim, Switzerland)	Methanol
	7632-10-2	$C_{10}H_{15}N$ (**d,l-Methamphetamine**) (MW=149.24 g/mol)		
**Moxifloxacin**	186826-86-8	$C_{21}H_{25}ClFN_3O_4$ (**Moxifloxacin hydrochloride**) (MW=437.90 g/mol)	Sigma-Aldrich (Madrid, Spain)	Ultrapure Water-10% acetic acid in ultrapure water (1:1, v/v)
	151096-09-2	$C_{21}H_{24}FN_3O_4$ (**Moxifloxacin**) (MW=401.44 g/mol)		
**Naproxen**	22204-53-1	$C_{14}H_{14}O_3$ (MW=230.26 g/mol)	Sigma-Aldrich (Madrid, Spain)	Acetonitrile-metanol (1:1,v/v)
**Nimesulide**	51803-78-2	$C_{13}H_{12}N_2O_5S$ (MW=308.31 g/mol)	Sigma-Aldrich (Madrid, Spain)	Acetonitrile
**d,l-Norephedrine**	154-41-6	$C_9H_{14}ClNO$ (**d,l-Norephedrine hydrochloride**) (MW=187.67 g/mol)	Lipomed AG (Arlesheim, Switzerland)	Methanol
	37577-28-9	$C_9H_{13}NO$ (MW=151.21 g/mol)		
**Norfloxacin**	70458-96-7	$C_{16}H_{18}FN_3O_3$ (MW=319.33 g/mol)	Sigma-Aldrich (Madrid, Spain)	Ultrapure Water-10% acetic acid in ultrapure water (1:1, v/v)
**Norfluoxetine**	57226-68-3	$C_{16}H_{17}ClF_3NO$ (**Norfluoxetine hydrochloride)** (MW=331.76 g/mol)	Sigma-Aldrich (Madrid, Spain)	Methanol
	83891-03-6	$C_{16}H_{16}F_3NO$ (MW=295.31 g/mol)		
**Norsertraline**	675126-08-6	$C_{16}H_{16}Cl_3N$ (**Norsertraline hydrochloride** (MW=328.66 g/mol)	Cerilliant-Certified Reference Materials (Texas, USA)	Purchased as methanolic solution
	87857-41-8	$C_{16}H_{15}Cl_2N$ (MW=292.20 g/mol)		
**O-desmethylvenlafaxine**	93413-62-8	$C_{16}H_{25}NO_2$ (MW=263.38 g/mol)	Sigma-Aldrich (Madrid, Spain)	Purchased as methanolic solution
**Ofloxacin**	82419-36-1	$C_{18}H_{20}FN_3O_4$ (MW=361.37 g/mol)	Sigma-Aldrich (Madrid, Spain)	Ultrapure Water-10% acetic acid in ultrapure water (1:1, v/v)

(cont Table S2)

Pharmaceuticals, metabolites, degradation product, and Isotopically Labelled Internal Standards[Note 1]	CAS [Note 2,3]	Formula (Molecular Weight) [Note 3]	Supplier Company	Solvent used for the preparation of each stock solution
**Oxytetracycline**	2058-46-0	$C_{22}H_{25}ClN_2O_9$ (**Oxytetracycline hydrochloride**) (MW=496.90 g/mol)	Sigma-Aldrich (Madrid, Spain)	Acetonitrile-metanol (1:1, v/v)
	2058-46-0	$C_{22}H_{24}N_2O_9$ (**Oxytetracycline**) (MW=460.44 g/mol)		
**Paroxetine**	78246-49-8	$C_{19}H_{21}ClFNO_3$ (**Paroxetine hydrochloride**) (MW=365.83 g/mol)	Sigma-Aldrich (Madrid, Spain)	Methanol
	110429-35-1	$C_{19}H_{20}FNO_3$ (**Paroxetine**) (MW=329.37 g/mol)		
**Phenolphthalein**	77-09-8	$C_{20}H_{14}O_4$ (MW=318.33 g/mol)	Sigma-Aldrich (Madrid, Spain)	Methanol
**Phentermine**	1197-21-3	$C_{10}H_{16}ClN$ (**Phentermine hydrochloride**) (MW=185.70 g/mol)	Lipomed AG (Arlesheim, Switzerland)	Methanol
	122-09-8	$C_{10}H_{15}N$ (**Phentermine**) (MW=149.24 g/mol)		
**Pramipexole**	191217-81-9	$C_{10}H_{17}N_3S$ 2HCl H_2O (**Pramipexole dihydrochloride monohydrate**) (MW=302.26 g/mol)	Sigma-Aldrich (Madrid, Spain)	Ultrapure water
	104632-26-0	$C_{10}H_{17}N_3S$ (**Pramipexole**) (MW=211.33 g/mol)		
**Pravastatin**	81131-70-6	$C_{23}H_{35}NaO_7$ (**Pravastatin sodium salt hydrate**) (MW=446.52 g/mol)	Sigma-Aldrich (Madrid, Spain)	Methanol
	81093-37-0	$C_{23}H_{36}O_7$ (**Pravastatin**) (MW=424.53 g/mol)		
**Propranolol**	318-98-9	$C_{16}H_{22}ClNO_2$ (**Propranolol hydrochloride**) (MW=295.81 g/mol)	Sigma-Aldrich (Madrid, Spain)	Methanol
	525-66-6	$C_{16}H_{21}NO_2$ (**Propranolol**) (MW=259.35 g/mol)		
**Prulifloxacin**	123447-62-1	$C_{21}H_{20}FN_3O_6S$ (MW=461.46 g/mol)	Toronto Research Chemicals Inc. (Ontario, Canada)	Ultrapure Water-10% acetic acid in ultrapure water (1:1, v/v)
**Rasagiline**	161735-79-1	$C_{12}H_{13}N$ CH_4O_3S (**Rasagiline mesylate**) (MW=267.34 g/mol)	Sigma-Aldrich (Madrid, Spain)	Ultra-pure water
	136236-51-6	$C_{12}H_{13}N$ (**Rasagiline**) (MW=171.24 g/mol)		
**Rivastigmine**	129101-54-8	$C_{14}H_{22}N_2O_2$ $C_4H_6O_6$ (**Rivastigmine hydrogen tartrate**) (MW=400.42 g/mol)	Sigma-Aldrich (Madrid, Spain)	Ultra-pure water
	123441-03-2	$C_{14}H_{22}N_2O_2$ (**Rivastigmine**) (MW=250.34 g/mol)		
**Ropinirole**	91374-20-8	$C_{16}H_{24}N_2O$ HCl (**Ropinirole hydrochloride**) (MW=296.84 g/mol)	Sigma-Aldrich (Madrid, Spain)	Ultra-pure water
	91374-21-9	$C_{16}H_{24}N_2O$ (**Ropinirole**) (MW=260.38 g/mol)		
**Rotigotine**	125572-93-2	$C_{19}H_{25}NOS \cdot HCl$ (**Rotigotine hydrochloride**) (MW=351.93 g/mol)	Sigma-Aldrich (Madrid, Spain)	Ethanol
	99755-59-6	$C_{19}H_{25}NOS$ (**Rotigotine**) (MW 315.48 g/mol)		
**Rimonabant**	168273-06-1	$C_{22}H_{21}Cl_3N_4O$ (MW=463.79 g/mol)	LGC (Middlesex, UK)	Methanol
**Safinamide**	202825-46-5	$C_{17}H_{19}FN_2O_2$ xCH_4O_3S (**Safinamide mesylate salt**) (MW=302.34 g/mol)	Sigma-Aldrich (Madrid, Spain)	Ultra-pure water
	133865-89-1	$C_{17}H_{19}FN_2O_2$ (**Safinamide**) (MW=302.34 g/mol)		
**Salicylic acid**	69-72-7	$C_7H_6O_3$ (MW=138.12 g/mol)	Sigma-Aldrich (Madrid, Spain)	Acetonitrile
**Sertraline**	79559-97-0	$C_{17}H_{18}Cl_3N$ (**Sertraline hydrochloride**) (MW=342.69 g/mol)	Sigma-Aldrich (Madrid, Spain)	Methanol
	79617-96-2	$C_{17}H_{17}Cl_2N$ (**Sertraline**) (MW=306.23 g/mol)		
**Sibutramine**	84485-00-7	$C_{17}H_{27}Cl_2N$ (**Sibutramine hydrochloride**) (MW=316.31 g/mol)	LGC (Middlesex, UK)	Purchased as methanolic solution
	106650-56-0	$C_{17}H_{26}ClN$ (**Sibutramine**) (MW=279.85 g/mol)		
**Simvastatin**	79902-63-9	$C_{25}H_{38}O_5$ (MW=418.57 g/mol)	Sigma-Aldrich (Madrid, Spain)	Methanol
**Sulfadiazine**	68-35-9	$C_{10}H_{10}N_4O_2S$ (MW=250.28 g/mol)	Sigma-Aldrich (Madrid, Spain)	Methanol+5µL NaOH+5 µL HCL
**Sulfadimethoxine**	122-11-2	$C_{12}H_{14}N_4O_4S$ (MW=310.33 g/mol)	Sigma-Aldrich (Madrid, Spain)	Methanol
**Sulfamethazine**	57-68-1	$C_{12}H_{14}N_4O_2S$ (MW=278.33 g/mol)	Sigma-Aldrich (Madrid, Spain)	Methanol
**Sulfamethizole**	144-82-1	$C_9H_{10}N_4O_2S_2$ (MW=270.33 g/mol)	Sigma-Aldrich (Madrid, Spain)	Acetonitrile-metanol (1:1, v/v)
**Sulfamethoxazole**	723-46-6	$C_{10}H_{11}N_3O_3S$ (MW=253.28 g/mol)	Sigma-Aldrich (Madrid, Spain)	Methanol
**Sulfamethoxypyridazine**	80-35-3	$C_{11}H_{12}N_4O_3S$ (MW=280.30 g/mol)	Sigma-Aldrich (Madrid, Spain)	Methanol
**Sulfapyridine**	144-83-2	$C_{11}H_{11}N_3O_2S$ (MW=249.29 g/mol)	Sigma-Aldrich (Madrid, Spain)	Methanol
**Sulfaquinoxaline**	59-40-5	$C_{14}H_{12}N_4O_2S$ (MW=300.34 g/mol)	Sigma-Aldrich (Madrid, Spain)	Methanol
**Sulfathiazole**	144-74-1	$C_9H_8N_3NaO_2S_2$ (**Sulfathiazole sodium salt**) (MW=277.29 g/mol)	Sigma-Aldrich (Madrid, Spain)	Acetonitrile-metanol (1:1, v/v)
	72-14-0	$C_9H_9N_3O_2S_2$ (**Sulfathiazole**) (MW=255.31 g/mol)		
**Synephrine**	94-07-5	$C_9H_{13}NO_2$ (MW= 167.21 g/mol)	LGC (Middlesex, UK)	Methanol
**Tetracycline**	64-75-5	$C_{22}H_{25}ClN_2O_8$ (**Tetracycline hydrochloride**) (MW=480.90 g/mol)	Sigma-Aldrich (Madrid, Spain)	Acetonitrile-metanol (1:1, v/v)
	64-75-5	$C_{22}H_{24}N_2O_8$ (**Tetracycline**) (MW=444.44 g/mol)		
**Topiramate**	97240-79-4	$C_{12}H_{21}NO_8S$ (MW=339.36 g/mol)	Lipomed AG (Arlesheim, Switzerland)	Methanol
**Trazodone**	19666-36-5	$C_{19}H_{23}Cl_2N_5O$ (**Trazodone hydrochloride**) (MW=408.33 g/mol)	Sigma-Aldrich (Madrid, Spain)	Methanol
	19794-93-5	$C_{19}H_{22}ClN_5O$ (**Trazodone**) (MW=371.869 g/mol)		

(cont Table S2)

Pharmaceuticals, metabolites, degradation product, and Isotopically Labelled Internal Standards[Note 1]	CAS [Note 2,3]	Formula (Molecular Weight) [Note 3]	Supplier Company	Solvent used for the preparation of each stock solution
**Trimethoprim**	738-70-5	$C_{14}H_{18}N_4O_3$ (MW=290.32 g/mol)	Sigma-Aldrich (Madrid, Spain)	Methanol
**Venlafaxine**	99300-78-4	$C_{17}H_{28}ClNO_2$ (**Venlafaxine hydrochloride**) (MW=313.87 g/mol)	Sigma-Aldrich (Madrid, Spain)	Methanol
	93413-69-5	$C_{17}H_{27}NO_2$ (**Venlafaxine**) (MW=277.41 g/mol)		
**Zonisamide**	68291-97-4	$C_8H_8N_2O_3S$ (MW=212.22 g/mol)	LGC (Middlesex, UK)	Methanol
**Acetaminophen-d4**	64315-36-2	$C_8H_5D_4NO_2$ (MW=155.189 g/mol)	Toronto Research Chemicals Inc. (Ontario, Canada)	Acetonitrile
**Azithromycin-d3**	163921-65-1	$C_{38}H_{69}D_3N_2O_{12}$ (MW=752.014 g/mol)	Toronto Research Chemicals Inc. (Ontario, Canada)	Methanol
**Caffeine $^{13}C_3$**	78072-66-9	$^{13}C_3C_5H_{10}N_4O_2$ (MW=197.17 g/mol)	Sigma-Aldrich (Madrid, Spain)	Purchased as methanolic solution
**Carbamazepine-d10**	132183-78-9	$C_{15}H_2D_{10}N_2O$ (MW=246.33 g/mol)	Cerilliant-Certified Reference Materials (Texas, USA)	Purchased as methanolic solution
**d,l-Methamphetamine-d5 hydrochloride**	60124-88-1	$C_{10}H_{11}ClD_5N$ (MW=190.74 g/mol)	Lipomed AG (Arlesheim, Switzerland)	Methanol
**Diazepam-d5**	65854-76-4	$C_{16}H_8D_5ClN_2O$ (MW=289.77 g/mol)	Lipomed AG (Arlesheim, Switzerland)	Purchased as methanolic solution
**Fluoxetine-d5 hydrochloride**	1173020-43-3	$C_{17}H_{14}D_5ClF_3NO$ (MW=350.821 g/mol)	Sigma-Aldrich (Madrid, Spain)	Methanol
**Gemfibrozil-d6**	1184986-45-5	$C_8H_5D_4NO_2$ (MW=256.37 g/mol)	Toronto Research Chemicals Inc. (Ontario, Canada)	Methanol
**Ibuprofen-d3**	121662-14-4	$C_{13}H_{18}D_3O_2$ (MW=209.30 g/mol)	Sigma-Aldrich (Madrid, Spain)	Acetonitrile
**Metformin-(dimethyl-d6) hydrochloride**	1185166-01-1	$C_4D_6H_6ClN_5$ (MW=171.66 g/mol)	Sigma-Aldrich (Madrid, Spain)	Methanol
**Salicylic acid-d4**	97781-16-3	$C_9H_4D_4O_4$ (MW=184.18 g/mol)	Toronto Research Chemicals Inc. (Ontario, Canada)	Acetonitrile
**Sulfamethoxazole-d4**	1020719-86-1	$C_{10}H_7D_4N_3O_3S$ (MW=257.3 g/mol)	Toronto Research Chemicals Inc. (Ontario, Canada)	Methanol
**Topiramate-d12**	1279037-95-4	$C_{12}H_9D_{12}NO_8S$ (MW=351.44 g/mol)	LGC (Middlesex, UK)	Purchased as methanolic solution
**Venlafaxine-d6**	1062606-12-5	$C_{17}H_{22}ClD_6NO_2$ (MW=319.90 g/mol)	Cerilliant-Certified Reference Materials (Texas, USA)	Purchased as methanolic solution

* the information of CAS is not available for the compound citalopram propionic acid.

Note 1: Pharmaceuticals organized in the table by alphabetic order.

Note 2: Chemical Abstracts Service, CAS (a unique numerical identifier assigned by the Chemical Abstracts Service to every chemical substance described in the open scientific literature).

Note 3: CAS, formula, and molecular weight for all the compounds were obtained in the web site of PubChem (PubChem is an open chemistry database at the National Institutes of Health (NIH) [Available at: https://pubchem.ncbi.nlm.nih.gov/], with exception for citalopram propionic acid [Available at: http://www.hmdb.ca/metabolites/HMDB60463].

Table S3. Therapeutic class, pharmaceuticals, ionization mode, precursor ions, product ions, mass spectrometry conditions, ion ratio, and isotopically labeled internal standards (ILIS) for each pharmaceutical in the study and chromatographic program.

**Therapeutic class and Pharmaceuticals, metabolites, degradation products, and isotopically labeled internal standards (ILIS)**		**ESI**	**Precursor (m/z)**	**Quantitation product**				**Qualifier Product**				**Dwell**	**ILIS**	Chromatographic Program
				m/z	***Q1***	***CE***	***Q3***	***m/z***	***Q1***	***CE***	***Q3***			
					Pre Bias (V)		***Pre Bias (V)***		***Pre Bias (V)***		***Pre Bias (V)***	**Time (msec)**		
Analgesic	Acetaminophen	ESI-	150.20	107.15	16	20	17	Note 1				25	2	PI
NSAIDs	Acetylsalicylic acid	ESI-	179.30	137.15	20	10	21	92.90	19	22	10	25	1	PI
NSAIDs	Ibuprofen	ESI-	205.20	161.30	21	10	23	Note 1				25	3	PI
NSAIDs	Ketoprofen	ESI-	253.20	209.20	27	7	12	Note 1				25	3	PI
NSAIDs-metabolite	2-Hydroxyibuprofen	ESI-	221.20	177.30	23	8	10	Note 1				25	3	PI
NSAIDs	Naproxen	ESI-	229.20	170.00	24	16	17	169.25	25	31	17	25	3	PI
NSAIDs	Nimesulide	ESI-	307.00	229.15	15	16	17	79.05	15	26	29	25	3	PI
NSAIDs-degradation product	Salicylic acid	ESI-	137.30	93.10	15	17	14	65.10	15	30	10	25	1	PI
NSAIDs-metabolite	Carboxyibuprofen	ESI-	235.20	191.25	25	9	16	72.90	24	17	11	25	3	PI
NSAIDs	Diclofenac	ESI-	294.10	250.20	11	11	14	35.05	14	24	11	25	3	PI
Antibiotic	Ampicillin	ESI+	348.00	307.05	-17	-10	-21	Note 1				15	12	PII
β-blockers	Atenolol	ESI+	267.05	144.95	-10	-28	-15	56.00	-22	-34	-11	15	12	PII
Lipid and cholesterol regulator statin drug	Atorvastatin	ESI+	559.10	440.05	-20	-24	-16	250.00	-20	-47	-16	15	12	PII
Antibiotic	Azithromycin	ESI+	749.30	158.00	-28	-45	-16	116.10	-38	-55	-11	10	9	PII
Stimulant	Caffeine	ESI+	194.95	138.00	-22	-21	-13	42.00	-23	-41	-16	15	8	PII
Pshychiatric drug	Carbamazepine	ESI+	236.95	193.95	-18	-21	-23	193.00	-26	-36	-13	10	11	PII
Antibiotic	Chlorocycline	ESI+	479.00	444.00	-24	-24	-15	462.00	-24	-20	-22	15	12	PII
Antipsychotic drug	Chlorpromazine	ESI+	319.00	85.95	-22	-21	-18	58.00	-25	-43	-12	15	12	PII
Antibiotic	Ciprofloxacin	ESI+	332.00	314.05	-17	-22	-22	231.00	-24	-39	-16	10	10	PII
Psychiatric drug	Citalopram	ESI+	325.05	108.95	-16	-29	-23	261.95	-16	-22	-18	10	12	PII
Psychiatric drug -metabolite	Citalopram N-oxide	ESI+	341.05	108.95	-17	-26	-10	261.95	-28	-19	-18	15	12	PII
Antibiotic	Clarithromycin	ESI+	748.30	158.05	-28	-34	-10	83.00	-28	-54	-17	10	9	PII
Psychiatric drug -metabolite	Desmethylcitalopram	ESI+	352.05	311.00	-29	-8	-15	108.95	-18	-34	-22	15	12	PII
Psychiatric drug	Diazepam	ESI+	284.95	153.90	-23	-29	-10	192.95	-23	-33	-23	10	7	PII
Psychiatric drug -metabolite	Didemethylcitalopram	ESI+	297.00	108.95	-25	-23	-10	262.00	-23	-15	-18	15	12	PII

(cont. Table S3)

**Therapeutic class and Pharmaceuticals, metabolites, degradation products, and isotopically labeled internal standards (ILIS)**		**ESI**	**Precursor (m/z)**	**Quantitation product**				**Qualifier Product**				**Dwell**	**ILIS**	Chromatographic Program
				m/z	***Q1***	***CE***	***Q3***	***m/z***	***Q1***	***CE***	***Q3***			
					Pre Bias		***Pre Bias***		***Pre Bias***		***Pre Bias***	**Time**		
					(V)		***(V)***		***(V)***		***(V)***	**(msec)**		
Calcium channel blocker	Diltiazem	ESI+	415.05	178.00	-20	-30	-20	150.00	-13	-48	-15	15	12	PII
Antibiotic	Doxycycline	ESI+	445.05	428.05	-17	-21	-15	97.95	-17	-47	-20	15	12	PII
Antibiotic	Enrofloxacin	ESI+	360.00	316.10	-18	-21	-15	342.10	-27	-23	-24	10	10	PII
Psychiatric drugs-metabolite	10,11-Epoxycarbamazepine	ESI+	253.00	179.95	-21	-27	-12	235.90	-21	-12	-16	10	11	PII
Antibiotic	Erythromycin	ESI+	734.40	158.00	-28	-33	-10	83.00	-28	-55	-19	10	9	PII
Fibrate lipid-lowering agent	Fenofibrate	ESI+	361.00	232.86	-29	-16	-16	138.95	-18	-35	-13	15	12	PII
Psychiatric drugs	Fluoxetine	ESI+	309.95	44.00	-25	-14	-18	Note 1				10	12	PII
Proton pump inhibitor	Lansoprazole	ESI+	370.00	251.90	-19	-13	-17	118.90	-30	-17	-12	15	12	PII
Antibiotic	Lomefloxacin	ESI+	352.00	264.95	-29	-25	-19	333.95	-18	-22	-24	10	10	PII
Antidiabetic drug	Metformin	ESI+	129.50	70.95	-10	-24	-14	59.95	-10	-15	-13	15	14	PII
Antibiotic	Moxifloxacin	ESI+	402.05	384.05	-21	-23	-26	95.95	-15	-48	-20	10	10	PII
Antibiotic	Norfloxacin	ESI+	320.00	302.05	-27	-22	-21	231.00	-26	-43	-16	10	10	PII
Psychiatric drug -metabolite	Norfluoxetine	ESI+	296.00	134.00	-23	-8	-13	30.20	-24	-15	-12	10	12	PII
Psychiatric drug -metabolite	Norsertraline	ESI+	291.90	274.90	-23	-11	-19	158.95	-22	-24	-10	10	12	PII
Psychiatric drug -metabolite	O-desmethylvenlafaxine	ESI+	264.00	58.00	-20	-25	-20	246.05	-21	-13	-17	15	13	PII
Antibiotic	Ofloxacin	ESI+	362.00	318.10	-29	-21	-15	260.95	-29	-29	-18	10	10	PII
Antibiotic	Oxytetracycline	ESI+	460.95	425.95	-23	-20	-15	443.05	-17	-15	-22	15	12	PII
Psychiatric drug	Paroxetine	ESI+	330.00	70.00	-27	-34	-14	44.00	-37	-28	-14	10	12	PII
β-blocker	Propanolol	ESI+	301.00	260.00	-25	-8	-18	Note 1				15	12	PII
Antibiotic	Prulifloxacin	ESI+	462.00	443.95	-17	-22	-22	360.00	-24	-32	-24	10	10	PII
Psychiatric drug	Sertraline	ESI+	305.95	158.90	-15	-26	-15	274.95	-25	-13	-19	10	12	PII
Lipid and cholesterol regulator statin drugs	Simvastatin	ESI+	419.15	199.00	-15	-15	-13	285.00	-16	-13	-20	15	12	PII
Antibiotic	Sulfadiazine	ESI+	250.90	155.90	-18	-18	-10	91.95	-19	-30	-19	10	10	PII
Antibiotic	Sulfadimethoxine	ESI+	310.90	155.90	-24	-23	-15	91.95	-25	-38	-18	10	10	PII
Antibiotic	Sulfamethazine	ESI+	278.90	185.90	-23	-19	-12	91.95	-22	-36	-19	10	10	PII
Antibiotic	Sulfamethizole	ESI+	270.85	155.95	-13	-16	-19	92.00	-21	-31	-19	15	10	PII
Antibiotic	Sulfamethoxazole	ESI+	253.95	155.90	-29	-18	-16	91.95	-20	-32	-18	10	10	PII

(cont. Table S3)

**Therapeutic class and Pharmaceuticals, metabolites, degradation products, and isotopically labeled internal standards (ILIS)**		**ESI**	**Precursor (m/z)**	**Quantitation product**				**Qualifier Product**				**Dwell**	**ILIS**	Chromatographic Program
				m/z	***Q1***	***CE***	***Q3***	***m/z***	***Q1***	***CE***	***Q3***			
					Pre Bias		***Pre Bias***		***Pre Bias***		***Pre Bias***	**Time**		
					(V)		***(V)***		***(V)***		***(V)***	**(msec)**		
Antibiotic	Sulfamethoxypyridazine	ESI+	280.90	155.95	-14	-19	-10	92.00	-14	-32	-18	10	10	PII
Antibiotic	Sulfapyridine	ESI+	249.90	155.95	-18	-18	-16	91.95	-28	-31	-20	10	10	PII
Antibiotic	Sulfaquinoxaline	ESI+	300.90	155.95	-25	-19	-10	92.00	-25	-37	-18	15	10	PII
Antibiotic	Sulfathiazole	ESI+	255.85	155.90	-19	-17	-16	92.00	-19	-29	-18	15	10	PII
Antibiotic	Tetracycline	ESI+	445.05	410.00	-13	-22	-20	153.95	-22	-29	-15	15	12	PII
Psychiatric drug	Trazodone	ESI+	372.05	175.95	-19	-26	-11	147.95	-19	-40	-15	10	12	PII
Antibiotic	Trimethoprim	ESI+	291.00	230.00	-23	-25	-15	123.00	-23	-27	-12	10	10	PII
Psychiatric drugs	Venlafaxine	ESI+	278.10	58.00	-20	-25	-20	260.05	-23	-14	-12	10	13	PII
Antibiotic	Amoxicillin	ESI-	364.10	223.00	17	11	12	205.95	18	18	28	25	5	PIII
Psychiatric drugs- metabolite	Citalopram propionic acid	ESI-	310.05	266.10	15	11	11	236.20	30	18	10	25	5	PIII
Lipid and cholesterol regulator statin drug	Gemfibrozil	ESI-	249.20	120.95	26	13	11	105.95	27	50	15	25	5	PIII
Stimulant, anorectic, anxiolytic, laxative	Phenolphthalein	ESI-	317.20	93.10	30	14	10	273.20	28	18	15	25	4	PIII
Lipid and cholesterol loweringregulator statin drug	Pravastatin	ESI-	423.15	101.15	21	30	15	58.85	20	24	17	25	5	PIII
Antibiotic	Potassium clavulanate	ESI-	198.10	135.90	13	10	10	108.10	11	13	15	25	5	PIII
Stimulant, anorectic, anxiolytic, laxative	Topiramate	ESI-	338.10	78.05	21	31	13	95.85	16	25	10	25	4	PIII
Stimulant, anorectic, anxiolytic, laxative	Zonisamide	ESI-	211.10	118.95	22	15	18	147.20	22	11	11	25	4	PIII
Stimulant, anorectic, anxiolytic, laxative	Alprazolam	ESI+	308.50	280.90	-25	-29	-19	205.05	-12	-43	-14	15	7*	PIV
Stimulant, anorectic, anxiolytic, laxative	Anfepramone	ESI+	205.80	105.00	-24	-24	-20	100.00	-10	-25	-20	15	6	PIV
Stimulant, anorectic, anxiolytic, laxative	Bupropion	ESI+	239.90	184.00	-29	-14	-20	130.95	-27	-30	-12	15	6	PIV
Stimulant, anorectic, anxiolytic, laxative	Clobenzorex	ESI+	260.00	91.00	-17	-27	-18	119.00	-21	-17	-11	15	6	PIV
Stimulant, anorectic, anxiolytic, laxative	*d*-Cathine	ESI+	151.90	134.00	-17	-15	-13	116.95	-17	-20	-11	15	6	PIV

(cont. Table S3)

**Therapeutic class and Pharmaceuticals, metabolites, degradation products, and isotopically labeled internal standards (ILIS)**		**ESI**	**Precursor (m/z)**	**Quantitation product**				**Qualifier Product**				**Dwell**	**ILIS**	Chromatographic Program
				m/z	***Q1***	***CE***	***Q3***	***m/z***	***Q1***	***CE***	***Q3***			
					Pre Bias		***Pre Bias***		***Pre Bias***		***Pre Bias***	**Time**		
					(V)		***(V)***		***(V)***		***(V)***	**(msec)**		
Stimulant, anorecticanxiolytic, laxative	dl-Methamphetamine	ESI+	149.80	91.05	-11	-22	-17	119.10	-11	-17	-24	15	6	PIV
Stimulant, anorectic, anxiolytic, laxative	dl-Norephedrine	ESI+	151.80	134.10	-10	-15	-14	117.05	-10	-21	-23	15	6	PIV
Stimulant, anorectic, anxiolytic, laxative	(+)-Ephedrine	ESI+	165.90	148.00	-20	-15	-16	116.95	-18	-22	-11	15	6	PIV
Stimulant, anorectic, anxiolytic, laxative	Fenfluramine	ESI+	231.90	159.90	-27	-230	-10	109.00	-26	-48	-10	15	6	PIV
Stimulant, anorectic, anxiolytic, laxative	Lorazepam	ESI+	320.90	274.85	-26	-24	-18	302.90	-26	-17	-14	15	7*	PIV
Stimulant, anorectic, anxiolytic, laxative	Mazindol	ESI+	284.90	44.00	-23	-27	-17	Note 1				15	6	PIV
Stimulant, anorectic, anxiolytic, laxative	Phentermine	ESI+	149.90	90.95	-17	-22	-20	18.05	-17	-9	-20	15	6	PIV
Stimulant, anorectic, anxiolytic, laxative	Rimonabant	ESI+	462.90	362.90	-23	-30	-25	84.00	-23	-28	-18	15	6	PIV
Stimulant, anorectic, anxiolytic, laxative	Sibutramine	ESI+	279.90	124.90	-23	-26	-13	139.00	-19	-17	-14	15	6	PIV
Stimulant, anorectic, anxiolytic, laxative	Synephrine	ESI+	167.90	149.95	-19	-12	-18	90.95	-19	-23	-19	15	6	PIV
Used to treat Parkinson's disease	Amantadine	ESI+	151.95	135.05	-20	-20	-20	79.00	-13	-34	-16	10	12	PV
Used to treat Parkinson's disease	Apomorphine	ESI+	268.10	191.05	-20	-30	-20	237.00	-20	-20	-20	10	12	PV
Used to treat Parkinson's disease	Benserazide	ESI+	257.90	120.00	-20	-10	-20	103.00	-20	-20	-20	10	12	PV
Used to treat Parkinson's disease	Carbidopa	ESI+	227.00	181.20	-19	-14	-12	71.00	-18	-24	-13	10	12	PV
Used to treat Alzheimer's disease	Donepezil	ESI+	380.15	90.95	-20	-40	-20	243.15	-15	-28	-17	10	12	PV
Used to treat Alzheimer's disease	Galantamine	ESI+	288.00	213.00	-23	-24	-21	198.05	-23	-34	-13	10	12	PV
Used to treat Parkinson's disease	Pramipexole	ESI+	211.90	152.95	-20	-15	-20	111.05	-17	-29	-10	10	12	PV
Used to treat Parkinson's disease	R(-)-Deprenyl (Selegiline)	ESI+	188.00	91.00	-20	-25	-20	119.00	-20	-15	-20	10	12	PV
Used to treat Parkinson's disease	Rasagiline	ESI+	172.00	117.00	-20	-15	-20	56.00	-14	-7	-19	10	12	PV
Used to treat Alzheimer's and Parkinson's disease	Rivastigmine	ESI+	251.00	206.00	-20	-15	-20	86.00	-20	-25	-20	10	12	PV
Used to treat Parkinson's disease	Ropinirole	ESI+	261.15	114.05	-20	-20	-20	86.05	-20	-35	-20	10	12	PV

(cont. Table S3)

**Therapeutic class and Pharmaceuticals, metabolites, degradation products, and isotopically labeled internal standards (ILIS)**		**ESI**	**Precursor (m/z)**	**Quantitation product**				**Qualifier Product**				**Dwell**	**ILIS**	Chromatographic Program
				m/z	***Q1***	***CE***	***Q3***	***m/z***	***Q1***	***CE***	***Q3***			
					Pre Bias		***Pre Bias***		***Pre Bias***		***Pre Bias***	**Time**		
					(V)		***(V)***		***(V)***		***(V)***	**(msec)**		
Used to treat Parkinson's disease	Rotigotine	ESI+	315.15	147.00	-20	-25	-20	107.00	-11	-48	-20	10	12	PV
Used to treat Parkinson's disease	Safinamide	ESI+	302.95	215.00	-20	-10	-20	108.95	-20	-25	-20	10	12	PV
Used to treat Parkinson's disease	Entacapone ESI-	ESI-	304.05	66.05	15	22	10	184.00	15	31	28	75	3	PVI
ILIS-1	Salycilin acid-d4	ESI-	141	97.05	16	17	20	Note 2				25	-	PI
ILIS-2	Acetaminophen-d4	ESI-	154.1	111.05	20	20	20	Note 2				25	-	PI
ILIS-3	Ibuprofen-d3	ESI-	208	164	21	10	23	Note 2				25	-	PI, PVI
ILIS-4	Topiramate-d12	ESI-	350.2	77.95	21	31	13	Note 2				25	-	PIII
ILIS-5	Gemfibrazil-d6	ESI-	255.2	121.1	26	13	11	Note 2				25	-	PIII
ILIS-6	dl-Methamphetamine-d5	ESI+	154.7	92.1	-11	-21	-17	Note 2				15	-	PIV
ILIS-7	Diazepam-d5	ESI+	289.9	154.05	-23	-31	-10	Note 2				10	--	PII
ILIS-7	Diazepam-d5	ESI+	289.9	154.05	-23	-31	10	Note 2				15	-	PIV
ILIS-8	Caffeine 13C3	ESI+	197.95	140.05	-22	-22	-14	Note 2				10	-	PII
ILIS-9	Azithromycin-d3	ESI+	752.3	83.15	-38	-55	-11	Note 2				10	-	PII
ILIS-10	Sulfamethoxazole-d4	ESI+	258	96.1	-29	-18	-16	Note 2				10	-	PII
ILIS-11	Carbamazepine-d10	ESI+	246.95	204.1	-18	-22	-23	Note 2				10	-	PII
ILIS-12	Fluoxetine-d5	ESI+	315.05	44.05	-25	-15	-20	Note 2				10	-	PII, PV
ILIS-13	Venlafaxine-d6	ESI+	283.8	64.05	-20	-25	-20	Note 2				10	-	PII
ILIS-14	Metformin-d6	ESI+	135.95	60.1	-10	-16	-13	Note 2				10	-	PII

Note1 and Note 2- Only one transition was recorder

Table S4. Chromatographic conditions, eluents, mode of elution, and source-dependent parameters for each chromatographic program in the negative and positive ionization modes.

Chromatographic program	ESI mode	Chromatographic Conditions	Eluents	Mode of elution		Source dependent parameters
Program I	Negative	Column: Kinetex C18 column (2.6 x 150 mm i.d., 1.7 µm particle size) from Phenomenex, Inc. (California, USA).<br>Flow rate: 0.22 mL/min<br>Oven temperature=30ºC<br>Injection volume= 5 µL	Eluent A<br>Ultra-pure water<br><br>Eluent B<br>Acetonitrile	**Time (min)**<br>0.0<br>1.0<br>2.0<br>6.0<br>6.5<br>10.5	**%Acetonitrile**<br>30.0<br>35.6<br>100<br>100<br>30.0<br>30.0	NGF=2.6 L/min<br>DGF=12.5 L/min<br>IV=5.0 kV<br>DLT=250ºC<br>HBT=300ºC
Program II	Positive	Column: CortecsTM UPLC® C18+ column (100 x 2.1 mm i.d.; 1.6 µm particle size) from Waters (Milford, Massachusetts, USA)<br>Flow rate: 0.30 mL/min<br>Oven temperature=30ºC<br>Injection volume= 5 µL	Eluent A<br>0.1% formic acid in ultrapure water<br><br>Eluent B<br>acetonitrile	**Time (min)**<br>0.0<br>3.0<br>3.5<br>4.0<br>7.0	**%Acetonitrile**<br>5.0<br>100<br>100<br>5.0<br>5.0	NGF=2.6 L/min<br>DGF=15 L/min<br>IV=5.0 kV<br>DLT=300ºC<br>HBT=425ºC
Program III	Negative	Column: Kinetex C18 column (2.6 x 150 mm i.d., 1.7 µm particle size) from Phenomenex, Inc. (California, USA)<br>Flow rate: 0.30 mL/min<br>Oven temperature=30ºC<br>Injection volume= 5 µL	Eluent A<br>Ultra-pure water<br><br>Eluent B<br>Acetonitrile	**Time (min)**<br>0.0<br>5.5<br>6.5<br>7.0<br>9.0	**%Acetonitrile**<br>10<br>100<br>100<br>10<br>10	NGF=2.6 L/min<br>DGF=15 L/min<br>IV=5.0 kV<br>DLT=300ºC<br>HBT=425ºC
Program IV	Positive	Column: CortecsTM UPLC® C18+ column (100 x 2.1 mm i.d.; 1.6 µm particle size) from Waters (Milford, Massachusetts, USA)<br>Flow rate: 0.30 mL/min<br>Oven temperature=30ºC<br>Injection volume= 5 µL	Eluent A<br>0.1% formic acid in ultrapure water<br><br>Eluent B<br>acetonitrile	**Time (min)**<br>0.0<br>1.0<br>2.0<br>7.0<br>8.5<br>11.0	**%Acetonitrile**<br>5.0<br>5.0<br>100<br>100<br>5.0<br>5.0	NGF=2.6 L/min<br>DGF=15 L/min<br>IV=5.0 kV<br>DLT=300ºC<br>HBT=425ºC
Program V	Negative	Column: CortecsTM UPLC® C18+ column (100 x 2.1 mm i.d.; 1.6 µm particle size) from Waters (Milford, Massachusetts, USA)<br>Flow rate: 0.30 mL/min<br>Oven temperature=30ºC<br>Injection volume= 5 µL	Eluent A<br>Ultra-pure water<br><br>Eluent B<br>Acetonitrile	**Time (min)**<br>0.0<br>1.0<br>3.5<br>4.0<br>8.0	**%Acetonitrile**<br>5.0<br>100<br>100<br>5.0<br>5.0	NGF=2.6 L/min<br>DGF=15 L/min<br>IV=5.0 kV<br>DLT=300ºC<br>HBT=425ºC
Program VI	Positive	Column: CortecsTM UPLC® C18+ column (100 x 2.1 mm i.d.; 1.6 µm particle size) from Waters (Milford, Massachusetts, USA)<br>Flow rate: 0.30 mL/min<br>Oven temperature=30ºC<br>Injection volume= 5 µL	Eluent A<br>0.1% formic acid in ultrapure water<br><br>Eluent B<br>acetonitrile	**Time (min)**<br>0.0<br>3.0<br>3.5<br>4.0<br>8.0	**%Acetonitrile**<br>5.0<br>100<br>100<br>5.0<br>5.0	NGF=2.6 L/min<br>DGF=15 L/min<br>IV=5.0 kV<br>DLT=225ºC<br>HBT=425ºC

NGF-Nebulizing gas flow (nitrogen), **DGF**-Drying gas flow (nitrogen), **IV**-Interface voltage, **DLT**-Desolvation line temperature, and **HBT**-Heat block temperature.

Table S5. Method detection limits for each study compound for surface water and wastewater samples.

Compounds	**MDL (ng/L)**	**MDL (ng/L)**
	Surface Waters	Wastewaters
Acetaminophen	1.24	58.0
Acetylsalicylic acid	14.5	29.2
Alprazolam	0.040	0.700
Amantadine	0.700	0.700
Amoxicillin	0.420	2.80
Ampicillin	6.60	8.15
Anfepramone	0.160	3.00
Apormorphine	0.340	29.7
Atenolol	44.5	144
Atorvastatin	0.0400	0.800
Azithromycin	0.0400	6.65
Benserazide	12.9	42.3
Bupropion	0.280	0.0500
Caffeine	2.62	21.0
Carbamazepine	3.80	2.35
Carbidopa	14.3	43.8
Carboxyibuprofen	7.40	28.2
d-Cathine	5.12	13.2
Chlorocycline	41.3	146
Chlorpromazine	29.1	72.6
Ciprofloxacin	8.12	27.7
Citalopram	1.12	3.50
Citalopram demethyl	6.54	2.60
Citalopram didemethyl	1.42	17.5
Citalopram N-oxide	11.4	38.2
Citalopram propionic acid	20.3	75.4
Clarithromycin	0.0200	0.0500
Clobenzorex	0.700	0.800
O-desmethylvenlafaxine	1.12	5.10
Diazepam	0.370	5.70
Diclofenac	5.44	7.50
Diltiazem	0.0200	0.0500
Donepezil	7.62	26.1
Doxycycline	31.3	58.3
Enrofloxacin	10.9	48.6
Entacapone	8.14	55.5
Ephendrine	5.22	20.6
10,11-Epoxy carbamazepine	0.240	0.100
Erythromycin	0.300	5.45
Fenfluramine	2.88	6.25

(cont. Table S5)

Compounds	MDL (ng/L)	MDL (ng/L)
	Surface Waters	Wastewaters
Fenofibrate	6.84	84.6
Fentermine	2.28	6.05
Fluoxetine	0.120	0.0500
Galantamine	3.04	7.80
Gemfibrozil	0.620	1.40
2-Hydroxyibuprofen	6.80	1.95
Ibuprofen	1.76	46.7
Ketoprofen	7.58	1.50
Lanzoprazole	0.420	3.25
Lomefloxacin	3.68	10.2
Lorazepam	0.140	4.30
Mazindol	0.760	0.300
Metformin	0.060	0.250
dl-Methamphetamine	1.06	0.300
Moxifloxacin	1.56	6.85
Naproxen	2.14	5.00
Nimesulide	4.42	39.3
dl-Norephedrine	0.260	4.40
Norfloxacin	15.4	59.5
Norfluoxetine	0.260	1.00
Norsertraline	53.1	103
Ofloxacin	0.0800	0.400
Oxytetracycline	38.8	126
Paroxetine	5.14	29.3
Phenolphthalein	5.16	23.9
Potassium clavulanate	9.82	73.8
Pramipexole	6.46	44.1
Pravastatin	0.240	0.700
Propanolol	57.2	182
Prulifloxacin	0.660	0.590
Rasagiline	8.26	45.6
Rimonabant	0.280	2.80
Rivastignine	0.0400	0.800

(cont. Table S5)

Compounds	MDL (ng/L)	MDL (ng/L)
	Surface Waters	Wastewaters
Ropinirole	0.0200	0.400
Rotigotine	0.400	1.15
Safinamide	4.84	31.1
Salicylic acid	2.60	7.70
Selegiline	7.70	13.1
Sertraline	0.1000	0.0500
Sibutramine	0.520	0.650
Simvastatin	7.28	4.55
Sulfadiazine	0.120	0.300
Sulfadimethoxine	0.740	2.40
Sulfamethazine	0.0400	0.400
Sulfamethizole	0.200	0.300
Sulfamethoxazole	3.74	13.5
Sulfamethoxypyridazine	4.10	14.0
Sulfapyridine	5.22	0.900
Sulfaquinoxaline	6.58	47.3
Sulfathiazole	0.240	7.40
Synephrine	1.34	19.0
Tetracycline	19.4	78.3
Topiramate	0.060	0.250
Trazodone	0.420	0.800
Trimethoprim	0.140	0.200
Venlafaxine	0.360	1.15
Zonisamide	0.160	4.40

Table S6. Concentrations (ng/L) of each studied compound in surface waters and WWTP wastewaters during the 2022 and 2023 sampling campaigns.

Compounds	Concentration (ng/L)								
	2022			2023					
	Surface water	Wastewater		Surface water			Wastewater		
	R1 downstream	WWTP 1 Influent	WWTP 1 Efluent	R1 upstream	R1 downstream	R2 downstream	WWTP 1 Effluent	WWTP 2 Effluent	WWTP 3 Effluent
Acetaminophen	<MDL	3638	<MDL	<MDL	<MDL	<MDL	<MDL	<MDL	<MDL
Acetylsalicylic acid	n.d.	n.d.	n.d.	n.d.	n.d.	n.d.	n.d.	n.d.	n.d.
Alprazolam	133	105	114	12.4	35.2	n.d.	67.2	81.0	43.9
Anfepramone	n.d.	n.d.	n.d.	n.d.	n.d.	<MDL	n.d.	n.d.	n.d.
Amoxicillin	n.d.	n.d.	n.d.	n.d.	n.d.	n.d.	n.d.	n.d.	n.d.
Ampicillin	<MDL	<MDL	<MDL	<MDL	82.9	<MDL	<MDL	<MDL	209
Atorvastatin	1620	236	848	97.4	742	n.d.	1760	956	99.9
Atenolol	n.d.	n.d.	n.d.	n.d.	n.d.	n.d.	n.d.	n.d.	n.d.
Azithromycin	430	121	634	52.7	184	<MDL	773	798	46.7
Bupropion	44.2	<MDL	62.4	6.03	22.6	n.d.	<MDL	44.0	<MDL
Caffeine	3214	12,664	7805	654	1226	17.3	5703	952	425
Cathine	n.d.	n.d.	n.d.	n.d.	n.d.	n.d.	n.d.	n.d.	n.d.
Carbamazepine	981	177	1359	70.1	340	n.d.	900	563	250
Carboxyibuprofen	n.d.	7068	998	38.9	n.d.	n.d.	n.d.	n.d.	n.d.
Citalopram	162	34.3	218	18.9	54.4	n.d.	140	142	31.2
Citalopram demethyl	n.d.	n.d.	n.d.	n.d.	n.d.	n.d.	n.d.	n.d.	n.d.
Citalopram didemethyl	n.d.	n.d.	n.d.	n.d.	n.d.	n.d.	n.d.	n.d.	n.d.
Citalopram N-oxide	n.d.	n.d.	n.d.	n.d.	n.d.	n.d.	n.d.	n.d.	n.d.
Citalopram propionic acid	311	196	157	n.d.	n.d.	n.d.	n.d.	n.d.	n.d.
Ciprofloxacin	152	59.2	n.d.	n.d.	n.d.	n.d.	173	129	81.5
Clarithromycin	1.68	<MDL	15.2	<MDL	<MDL	n.d.	0.860	2.62	<MDL
Clobenzorex	n.d.	<MDL	n.d.	n.d.	n.d.	n.d.	n.d.	n.d.	n.d.
Chlorocycline	n.d.	n.d.	n.d.	n.d.	n.d.	n.d.	n.d.	n.d.	n.d.
Chlorpromazine	n.d.	n.d.	n.d.	n.d.	n.d.	n.d.	n.d.	n.d.	n.d.
Diazepam	<MDL	n.d.	n.d.	<MDL	<MDL	n.d.	n.d.	n.d.	n.d.
O-desmethylvenlafaxine	2304	639	2011	225	745	n.d.	1828	2124	2364
Diclofenac	4367	1164	4882	217	1060	n.d.	3132	1980	498
Diltiazem	16.4	<MDL	34.0	n.d.	<MDL	n.d.	<MDL	7.05	n.d.
Doxycycline	n.d.	n.d.	n.d.	n.d.	n.d.	n.d.	n.d.	n.d.	n.d.
10,11-epoxy carbamazepine	<MDL	<MDL	<MDL	n.d.	<MDL	n.d.	<MDL	<MDL	n.d.
Enrofloxacin	n.d.	n.d.	n.d.	n.d.	n.d.	n.d.	n.d.	n.d.	n.d.
Ephendrine	n.d.	n.d.	n.d.	n.d.	n.d.	n.d.	n.d.	n.d.	n.d.
Erythromycin	n.d.	n.d.	n.d.	n.d.	n.d.	n.d.	n.d.	n.d.	n.d.
Fenfluramine	n.d.	n.d.	n.d.	n.d.	n.d.	n.d.	n.d.	n.d.	n.d.
Fenofibrate	n.d.	n.d.	n.d.	n.d.	n.d.	n.d.	n.d.	n.d.	n.d.
Fentermine	n.d.	n.d.	n.d.	n.d.	n.d.	n.d.	n.d.	n.d.	n.d.
Fluoxetine	7.55	<MDL	34.4	<MDL	<MDL	<MDL	6.80	<MDL	<MDL
Gemfibrozil	19.2	13.3	15.2	5.82	19.9	2.53	60.6	75.7	10.3
2-Hydroxyibuprofen	5623	4215	7767	515	515	<MDL	1545	430	<MDL
Ibuprofen	2267	3733	4442	176	242	28.6	801	129	246
Ketoprofen	1338	891	1247	42.6	265	17.9	834	537	274
Lanzoprazole	n.d.	n.d.	n.d.	n.d.	n.d.	n.d.	n.d.	n.d.	n.d.
Lomefloxacin	n.d.	n.d.	n.d.	n.d.	n.d.	n.d.	n.d.	n.d.	n.d.
Lorazepam	n.d.	n.d.	n.d.	n.d.	n.d.	n.d.	n.d.	n.d.	n.d.
Mazindol	<MDL	<MDL	12.4	<MDL	<MDL	<MDL	<MDL	<MDL	<MDL
Metformin	n.d.	n.d.	n.d.	47.1	108	n.d.	92.0	n.d.	n.d.
Methamphetamine	n.d.	n.d.	n.d.	n.d.	n.d.	n.d.	n.d.	n.d.	n.d.
Moxifloxacin	n.d.	n.d.	n.d.	n.d.	n.d.	n.d.	n.d.	n.d.	n.d.

(cont Table S6)

**Compounds**	**Concentration (ng/L)**								
	2022			*2023*					
	**Surface water**	**Wastewater**		**Surface water**			**Wastewater**		
	**R1 downstream**	**WWTP 1 Influent**	**WWTP 1 Efluent**	**R1 upstream**	**R1 downstream**	**R2 downstream**	**WWTP 1 Effluent**	**WWTP 2 Effluent**	**WWTP 3 Effluent**
Naproxen	470	963	828	277	196	<MDL	404	122	287
Nimesulide	n.d.	n.d.	n.d.	n.d.	n.d.	n.d.	n.d.	n.d.	n.d.
Norsertraline	n.d.	n.d.	n.d.	n.d.	n.d.	n.d.	n.d.	n.d.	n.d.
Norfloxacin	n.d.	n.d.	n.d.	n.d.	n.d.	n.d.	n.d.	n.d.	n.d.
Norfluoxetine	n.d.	n.d.	n.d.	n.d.	n.d.	n.d.	n.d.	n.d.	n.d.
Norephedrine	n.d.	n.d.	n.d.	n.d.	n.d.	n.d.	n.d.	n.d.	n.d.
Ofloxacin	52.7	<MDL	167	n.d.	30.2	n.d.	57.2	74.4	48.0
Oxytetracycline	n.d.	n.d.	n.d.	n.d.	n.d.	n.d.	n.d.	n.d.	n.d.
Paroxetine	<MDL	n.d.	n.d.	n.d.	n.d.	n.d.	n.d.	<MDL	n.d.
Phenolphthalein	n.d.	n.d.	n.d.	n.d.	n.d.	n.d.	n.d.	n.d.	n.d.
Potassium clavulanate	n.d.	n.d.	n.d.	n.d.	n.d.	n.d.	n.d.	n.d.	n.d.
Pravastatin	n.d.	n.d.	n.d.	n.d.	n.d.	n.d.	n.d.	n.d.	n.d.
Propanolol	n.d.	n.d.	n.d.	n.d.	n.d.	n.d.	n.d.	n.d.	n.d.
Prulifloxacin	n.d.	n.d.	n.d.	n.d.	n.d.	n.d.	n.d.	n.d.	n.d.
Rimonabant	n.d.	n.d.	n.d.	n.d.	n.d.	n.d.	n.d.	n.d.	n.d.
Salicylic acid	175	8353	468	101	102	107	171	229	225
Sertraline	13.9	<MDL	166	<MDL	20.7	n.d.	57.0	<MDL	<MDL
Sibutramine	n.d.	n.d.	n.d.	n.d.	n.d.	n.d.	n.d.	n.d.	n.d.
Simvastatin	n.d.	n.d.	n.d.	n.d.	n.d.	n.d.	n.d.	n.d.	n.d.
Sulfadiazine	n.d.	n.d.	n.d.	n.d.	n.d.	n.d.	n.d.	n.d.	n.d.
Sulfadimethoxine	n.d.	n.d.	n.d.	n.d.	n.d.	n.d.	n.d.	n.d.	n.d.
Sulfaquinoxaline	n.d.	n.d.	n.d.	n.d.	n.d.	n.d.	n.d.	n.d.	n.d.
Sulfamethazine	n.d.	n.d.	n.d.	n.d.	n.d.	n.d.	n.d.	n.d.	n.d.
Sulfamethoxypyridazine	n.d.	n.d.	n.d.	n.d.	n.d.	n.d.	n.d.	n.d.	n.d.
Sulfamethoxazole	n.d.	n.d.	n.d.	n.d.	n.d.	n.d.	n.d.	n.d.	n.d.
Sulfamethizole	n.d.	n.d.	n.d.	n.d.	n.d.	n.d.	n.d.	n.d.	n.d.
Sulfapyridine	n.d.	n.d.	n.d.	n.d.	n.d.	n.d.	n.d.	n.d.	n.d.
Sulfathiazole	n.d.	n.d.	n.d.	n.d.	n.d.	n.d.	n.d.	n.d.	n.d.
Synephrine	n.d.	n.d.	n.d.	n.d.	n.d.	n.d.	n.d.	n.d.	n.d.
Venlafaxine	796	64.2	1029	51.1	234	n.d.	663	955	173
Trazodone	347	<MDL	503	<MDL	62.5	n.d.	201	94.0	<MDL
Tetracycline	n.d.	n.d.	n.d.	n.d.	n.d.	n.d.	n.d.	n.d.	n.d.
Trimethoprim	n.d.	<MDL	n.d.	15.1	50.7	<MDL	64.5	69.7	n.d.
Topiramate	392	n.d.	238	n.d.	n.d.	n.d.	264	323	156
Zonisamide	34.4	n.d.	n.d.	n.d.	n.d.	n.d.	n.d.	n.d.	n.d.
Amantadine	233	n.d.	n.d.	n.d.	14.1	n.d.	99.3	n.d.	97.7
Apormorphine	n.d.	n.d.	n.d.	n.d.	n.d.	n.d.	n.d.	n.d.	n.d.
Benserazide	n.d.	n.d.	n.d.	n.d.	n.d.	n.d.	n.d.	n.d.	n.d.
Carbidopa	n.d.	n.d.	n.d.	n.d.	n.d.	n.d.	n.d.	n.d.	n.d.
Donepezil	n.d.	n.d.	n.d.	n.d.	n.d.	n.d.	n.d.	n.d.	n.d.
Entacapone	n.d.	n.d.	n.d.	n.d.	n.d.	n.d.	n.d.	n.d.	n.d.
Glanthamine	n.d.	n.d.	n.d.	n.d.	n.d.	n.d.	n.d.	n.d.	n.d.
Pramipexole	n.d.	n.d.	n.d.	n.d.	n.d.	n.d.	n.d.	n.d.	n.d.
Rasagiline	n.d.	n.d.	n.d.	n.d.	n.d.	n.d.	n.d.	n.d.	n.d.
Rivastignine	n.d.	n.d.	n.d.	n.d.	n.d.	n.d.	17.0	18.1	n.d.
Ropinirole	n.d.	n.d.	n.d.	n.d.	n.d.	n.d.	n.d.	n.d.	n.d.
Rotigotine	n.d.	n.d.	n.d.	n.d.	n.d.	n.d.	n.d.	n.d.	n.d.
Safinamide	n.d.	n.d.	n.d.	n.d.	n.d.	n.d.	n.d.	n.d.	n.d.
Selegiline	n.d.	n.d.	n.d.	n.d.	n.d.	n.d.	n.d.	n.d.	n.d.

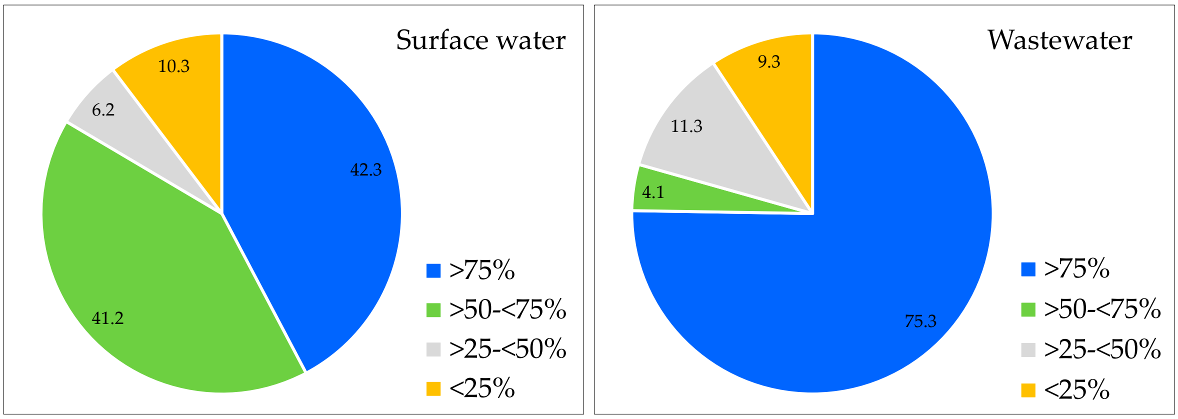

Figure S1. Percentage of recovered pharmaceuticals within each recovery range for surface water and wastewater.
